# Research progress on resistance mechanisms to CAR-T cell therapy in diffuse large B-cell lymphoma

**DOI:** 10.3389/fonc.2026.1696105

**Published:** 2026-02-19

**Authors:** Shuran Zhang, Jiye Liu, Zengjun Li

**Affiliations:** Department of Lymphoma, Shandong Cancer Hospital and Institute, Shandong First Medical University and Shandong Academy of Medical Sciences, Jinan, Shandong, China

**Keywords:** CAR (chimeric antigen receptor) T cell therapy2, DLBCL - diffuse large B cell lymphoma, immune evasion, molecular targeted drug, resistance mechanism, tumor microenvironment (TME)

## Abstract

Chimeric antigen receptor T-cell (CAR-T) therapy represents a revolutionary immunotherapy modality that has fundamentally transformed treatment paradigms for relapsed/refractory (r/r) hematological malignancies. For patients with r/r diffuse large B-cell lymphoma (DLBCL), CD19-targeted CAR-T cell therapy is currently approved in second-line and post-second-line settings, achieving substantial clinical responses in selected B-cell leukemia/lymphoma subgroups. Nevertheless, a significant proportion of B-cell lymphoma patients exhibit primary resistance or unsatisfactory long-term disease control after CAR-T infusion, substantially constraining therapeutic utility across both hematological and solid malignancies. Beyond the well-documented phenomenon of target antigen (CD19) loss, multifaceted resistance mechanisms against CAR-T therapy have been increasingly recognized. This review comprehensively explores potential resistance mechanisms in DLBCL through mechanistic insights from four interconnected dimensions: molecular alterations underlying tumor-associated CD19 expression loss; cell-intrinsic factors driving CAR-T cell differentiation arrest and functional exhaustion; immunomodulatory escape programs within the tumor microenvironment; and innate tumor cell resistance pathways. Elucidating these determinants provides critical foundations for developing novel therapeutic targets to overcome resistance. This knowledge promises to guide rational engineering of next-generation CAR-T cells with enhanced anti-tumor potency and reduced toxicity profiles, ultimately improving clinical outcomes across diverse malignancies.

## 1 Introduction

Diffuse large B-cell lymphoma (DLBCL), the most common pathological subtype of non-Hodgkin lymphoma, exhibits remarkable heterogeneity in its clinical presentation, molecular genetic background, and treatment response. This diversity stems not only from its complex molecular subtypes but also profoundly influences both therapeutic strategy development and patient prognosis. Traditionally, based on the cell-of-origin (COO) model, DLBCL is classified into germinal center B-cell-like (GCB) and activated B-cell-like (ABC) subtypes (3).

However, the traditional COO classification exhibits inherent limitations in identifying certain high-risk genetic subsets-{{-}}–such as “double-hit/triple-hit lymphomas” (DHL/THL)-{{-}}–prompting further refinement of DLBCL molecular subtyping. Advancements in precision medicine have fostered the evolution of classification systems grounded in molecular genetic characteristics, such as the five molecular subtypes (MCD, BN2, N1, EZB, A53) proposed by Schmitz et al (4). and the integrated C1-{{-}}-C5 classification framework established by Chapuy et al., which incorporates genomic architecture and immune microenvironmental features (5). These frameworks increasingly reveal the critical association between DLBCL’s molecular complexity-{{-}}–encompassing specific gene mutations and signaling pathway dysregulation-{{-}}–and clinical outcomes. Particularly, high-risk subtypes like DHL/THL involving rearrangements of MYC, BCL2, and/or BCL6, or double-expressor lymphoma (DEL) characterized by co-expression of MYC and BCL2 protein detected by immunohistochemistry, along with TP53 mutations, demonstrate five-year survival rates potentially below 30% (6–8). These entities constitute the challenging molecular foundation underlying current DLBCL treatment, especially for novel immunotherapies.

For decades, the R-CHOP regimen (rituximab, cyclophosphamide, doxorubicin, vincristine, and prednisone) has served as the cornerstone first-line therapy for diffuse large B-cell lymphoma (DLBCL). However, significant unmet clinical needs remain for patients with relapsed or refractory (R/R) disease, particularly those with high-risk molecular subtypes or those ineligible for transplantation. The advent of chimeric antigen receptor T-cell (CAR-T) therapy has fundamentally transformed the treatment landscape for R/R DLBCL, establishing itself as a pivotal option in the second-line and beyond. Long-term follow-up data confirm that anti-CD19 CAR-T therapy can induce durable deep responses in a subset of patients (1, 9).

Although CAR-T cell products have undoubtedly improved clinical outcomes and brought new hope for patients with refractory hematological malignancies, the practical experience since the first product’s approval nearly five years ago has also prompted significant reflection. Beyond the complexities of autologous cell manufacturing and the management of toxicities such as cytokine release syndrome (CRS), the most pressing challenge remains the suboptimal long-term disease control, manifested as either no response post-infusion (primary resistance) or relapse after initial remission (acquired resistance), which limits the broad application of this therapy in a substantial proportion of patients with B-cell malignancies. This resistance results from multifactorial interactions involving adaptive changes in tumor cells (e.g., CD19 antigen escape, activation of immune checkpoint molecules), dysfunctional states of CAR-T cells (such as terminal exhaustion, inadequate proliferation and persistence), and the immunosuppressive tumor microenvironment. Furthermore, a deeper understanding of the molecular basis underlying these resistance mechanisms will not only clarify the biological principles of CD19 CAR-T treatment failure but also provide critical insights and strategic directions for further optimization of cell-based immunotherapies.

Therefore, this review aims to systematically summarize recent research advances into the resistance mechanisms of diffuse large B-cell lymphoma (DLBCL) to chimeric antigen receptor T-cell (CAR-T) therapy, encompassing multiple dimensions including antigen escape, immunosuppressive tumor microenvironment, T-cell dysfunction, and tumor cell-intrinsic factors. It further discusses potential strategies to overcome resistance, along with relevant detection methods and predictive models. Through a systematic analysis of existing research findings, this work seeks to provide a theoretical foundation and novel perspectives for developing more effective CAR-T therapeutic strategies.

## 2 Multidimensional analysis of drug resistance mechanisms

### 2.1 Tumor cell-mediated resistance mechanisms

#### 2.1.1 Antigen escape

In diffuse large B-cell lymphoma (DLBCL), CD19 represents the preferred target for chimeric antigen receptor T-cell (CAR-T) therapy and is universally utilized in commercially available CAR-T products (10). Antigen escape constitutes one of the most prevalent and well-characterized mechanisms underlying treatment failure in CD19-targeted CAR-T therapy, implicated in approximately 30%-40% of patients with relapsed/refractory DLBCL (2). This phenomenon is driven by multifaceted molecular mechanisms, including genetic mutations, epigenetic silencing, alternative splicing, and disruptions in protein trafficking, which collectively establish the primary barrier to CAR-T efficacy in DLBCL.

##### 2.1.1.1 Genetic alterations and epigenetic silencing

At the genomic level, somatic mutations in the CD19 gene locus exhibit marked heterogeneity. Frameshift mutations in exon 2 (encoding the extracellular domain) and exon 4 (encoding the transmembrane domain) represent predominant genetic alterations, frequently resulting in truncated CD19 proteins or structural loss of antigenic epitopes (11, 12). Notably, biallelic inactivation—characterized by loss of heterozygosity (LOH) of one allele coupled with a loss-of-function mutation in the remaining allele—is detectable in a subset of relapsed DLBCL patients, leading to complete CD19 antigen loss (13, 14). Furthermore, hypermethylation of CpG islands within the CD19 promoter region serves as a crucial epigenetic regulatory mechanism. This aberrant methylation impedes the binding of key transcription factors such as PAX5, thereby substantially repressing CD19 transcriptional activity, a finding corroborated in tumor clones from relapsed DLBCL patients (15). It is noteworthy that while genomic alterations are often considered definitive molecular events, the prevalence of pre-existing mutations in treatment-naïve patients and their predictive value for treatment response require further validation in larger prospective cohorts. Most current evidence derives from post-relapse samples, which may be subject to selection bias.

##### 2.1.1.2 Transcriptional and post-transcriptional dysregulation

**Figure 1:**
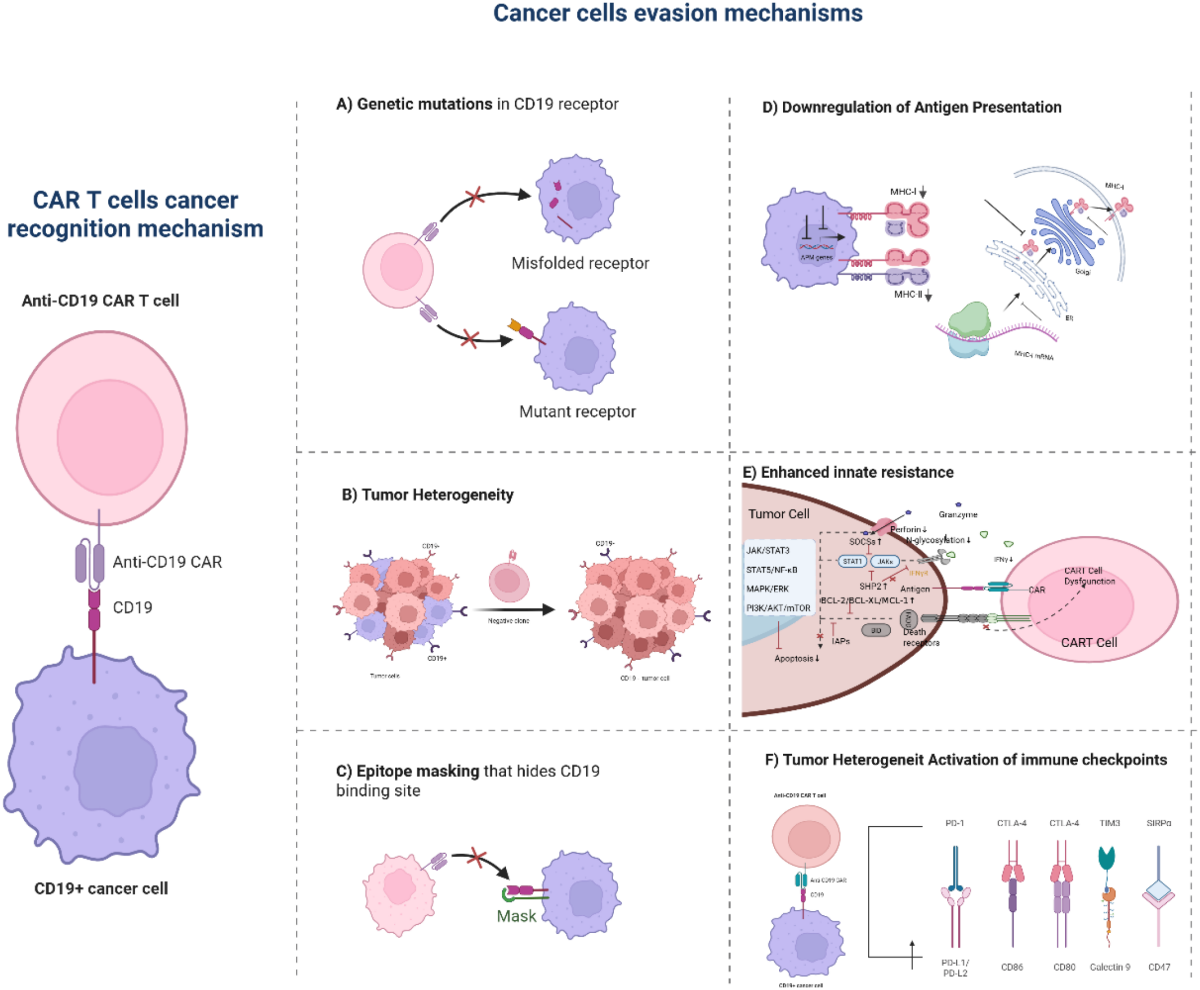
Anti-CD19 CAR-T cell recognition and cancer cell evasion mechanisms. This figure outlines how anti-CD19 CAR-T cells target CD19+ cancer cells (left panel: CARs on CAR-T cells bind CD19 on tumor cells) and six key cancer cell evasion strategies: **(A)** CD19 genetic mutations produce altered/misfolded receptors, disrupting CAR-T binding. **(B)** Tumor heterogeneity generates CD19− cancer cell subpopulations that avoid CAR-T recognition. **(C)** CD19 epitope masking conceals the CAR binding site on cancer cells. **(D)** Reduced MHC molecule expression impairs antigen presentation and immune surveillance. **(E)** Enhanced anti-apoptotic signaling/cytotoxic molecule resistance renders cancer cells refractory to CAR-T killing. **(F)** Upregulated immune checkpoint ligands (e.g., PD-L1) on cancer cells suppress CAR-T cell function. This illustration summarizes the foundational anti-CD19 CAR-T targeting mode and diverse cancer cell strategies driving resistance to this therapy.

At the level of mRNA processing, alternative splicing represents another critical pathway generating CD19-deficient variants. Key mechanisms include exon skipping and intron retention. For instance, skipping of exon 2 leads to complete absence of the encoded V-set Ig-like domain—which contains the critical epitope recognized by most scFvs (e.g., FMC63). Although these CD19 variants may retain some surface localization, their binding affinity to CAR-T cells is substantially diminished (14, 16, 17). Conversely, retention of intron 2 introduces a premature termination codon in the mRNA (9, 11), resulting in truncated protein products that typically misfold in the endoplasmic reticulum and undergo rapid degradation via the endoplasmic reticulum-associated degradation (ERAD) pathway, preventing effective membrane localization (17, 18) (**Figure 1A**). Emerging evidence further implicates post-transcriptional regulation in antigen escape: abnormal methylation of RNA-binding proteins (e.g., HuR) can accelerate CD19 mRNA decay (19), while the long non-coding RNA MALAT1 interferes with translational efficiency through R-loop formation (20, 21). Although splice variants are pivotal for “antigen-positive” escape, conventional flow cytometry may fail to distinguish full-length CD19 from variants lacking critical epitopes. This underscores the necessity of developing more precise antigen detection methodologies to accurately assess patient relapse risk.

##### 2.1.1.3 Defects in protein synthesis, maturation, and trafficking

Successful surface expression of CD19 relies on the integrity of the intracellular trafficking pathway from the endoplasmic reticulum (ER) and Golgi apparatus to the lysosome, and tumor cells can disrupt this process at multiple stages (22). Initially, within the ER, downregulation of key chaperone proteins such as CD81 can trigger aberrant activation of the unfolded protein response (UPR), leading to the retention and subsequent degradation of CD19 precursor proteins via the ER-associated degradation (ERAD) pathway (23). Concurrently, aberrant upregulation of interferon-induced transmembrane protein 1 (IFITM1) competitively inhibits the activity of oligosaccharyltransferase STT3A (24, 25), resulting in extensive loss of N-linked glycosylation on CD19. This modification defect directly disrupts the spatial conformation of the epitope recognized by CAR-T cells (26) (**Figure 1C**). Subsequently, in the Golgi apparatus, hyperactivation of protein kinase D compromises the function of sorting receptors, misrouting a significant fraction of CD19 molecules toward lysosomal destinations (27). Ultimately, within the lysosome, molecules such as lysosomal-associated transmembrane protein 5 (LAPTM5) and the splice variant LAMP2C act as “scavengers,” enabling precise clearance of residual CD19 proteins—LAPTM5 by enhancing ubiquitination-mediated degradation (28, 29) and LAMP2C via chaperone-mediated autophagy (30) (**Figure 1D**).

Collectively, these protein-level obstacles often act synergistically with upstream genetic and transcriptional abnormalities, creating a compounded effect that drives antigen escape. Notably, glycosylation defects in CD19 (26) represent a particularly stealthy mechanism of “antigen disguise,” where the physical presence of the antigen remains unchanged while its functionality is abolished. This underscores that antigen detection assays must extend beyond mere presence/absence quantification to assess the structural integrity and functional state of the target.

In summary, antigen escape constitutes a multi-step, multi-layered continuum. From DNA to mRNA and finally to functional membrane protein, tumor cells establish elaborate “checkpoints” at every stage to evade immune surveillance. A deeper understanding of this systematic network is crucial for guiding the development of next-generation CAR-T products—such as dual-targeted CARs or conformation-insensitive scFvs—and for rationally combining small-molecule inhibitors targeting protein trafficking pathways.

#### 2.1.2 Tumor heterogeneity and clonal evolution

Tumor heterogeneity represents one of the most fundamental biological characteristics of diffuse large B-cell lymphoma (DLBCL), creating a fertile breeding ground for variable responses to CAR-T therapy and the emergence of resistance. This heterogeneity exists not as a static entity but as a dynamic evolutionary process, continuously shaped by genomic instability, epigenetic reprogramming, and the selective pressures exerted by CAR-T cells themselves. It is particularly noteworthy that distinct molecular subtypes of DLBCL exhibit unique heterogeneity profiles, accompanied by divergent clonal evolutionary trajectories and mechanisms of resistance.

##### 2.1.2.1 Molecular subtype-specific heterogeneity blueprint

Molecular classification of DLBCL (such as the Cell-of-Origin subtypes and more refined genetic subtypes) provides a critical framework for understanding its heterogeneity. The germinal center B-cell-like (GCB) subtype is frequently associated with EZH2 mutations and BCL2 translocations (5), with its heterogeneity primarily manifesting at the epigenetic level. EZH2 overexpression leads to widespread deposition of H3K27me3 modifications, which not only silences the expression of B-cell identity genes like CD19 (31, 32) but also enhances chromatin accessibility for immune inhibitory molecules such as PD-L1, creating a so-called “epigenetic seesaw” effect (33). Conversely, the activated B-cell-like (ABC) subtype is characterized by constitutive activation of the NF-κB signaling pathway. Its heterogeneity is largely driven by mutations in genes like MYD88 and CD79B, which not only promote tumor cell survival but also contribute to therapy resistance by shaping a distinct tumor microenvironment (4, 6).

##### 2.1.2.2 Drivers of clonal evolution and selection of resistant subclones

The inherent high mutational burden and chromosomal instability in DLBCL provide the fundamental drivers for clonal evolution (34). Single-cell studies have confirmed the presence of CD19-negative or CD19-low tumor subclones even in treatment-naïve DLBCL patients (12). Under the selective pressure of CAR-T therapy, these pre-existing resistant subclones undergo preferential expansion through clonal selection, ultimately leading to treatment failure (**Figure 1B**). For instance, DNA editing activity mediated by the APOBEC3 family is recognized as a significant mechanism that further diversifies the tumor genome, generating novel resistant clones (35, 36). Furthermore, DHL/THL subtypes involving rearrangements of MYC, BCL2, and/or BCL6, due to their potent intrinsic anti-apoptotic capacity and high proliferative activity, inherently constitute “high-risk clones” with enhanced survival under therapeutic pressure, demonstrating relative insensitivity to various treatments, including CAR-T therapy (9, 11).

##### 2.1.2.3 Spatial heterogeneity and subclonal cooperation

Tumor heterogeneity manifests not only between cells but also in their spatial distribution. Spatial transcriptomic analyses reveal that distinct DLBCL subtypes construct unique immune microenvironment landscapes. For instance, according to the Chapuy classification, the C5 subtype, enriched with fibroblasts, forms a physical barrier, while the C1 subtype, characterized by significant T-cell infiltration alongside high exhaustion markers, constitutes a functional barrier (5). More complex still, different subclones can establish metabolic cooperation networks. Studies indicate that CD19-positive cells may support the survival of CD19-negative counterparts via signaling pathways such as IL-4/STAT6, while the negative subclones, in turn, promote aberrant angiogenesis through the secretion of factors like VEGF-A. This “symbiotic relationship” significantly diminishes the overall clearance efficiency of CAR-T cells (37–39).

Tumor heterogeneity implies that therapeutic strategies targeting a single antigen or pathway are inevitably circumvented by evolution. For example, in the EZB subtype, which is often enriched with pre-existing CD19-negative clones, even if CAR-T cells initially eliminate CD19-positive cells, the negative clones rapidly fill the void. This mechanistically explains why multi-targeting CAR-T strategies (e.g., CD19/CD22 dual-targeting CARs) demonstrate a theoretical and early clinical advantage in overcoming such resistance (40). Consequently, pre-therapeutic assessment of the tumor’s clonal architecture and heterogeneity degree via high-throughput sequencing holds crucial potential value for predicting CAR-T efficacy and guiding combination therapies.

In conclusion, tumor heterogeneity in DLBCL constitutes a multi-layered, dynamically evolving complex system. Different molecular subtypes, through their specific genomic, epigenetic, and microenvironmental features, shape distinct clonal evolutionary trajectories, ultimately giving rise to diverse modes of CAR-T resistance. Prospectively, dynamically mapping the “clonal evolution blueprint” of patients before and after therapy, based on single-cell and spatial multi-omics technologies, will be a fundamental prerequisite for achieving precision immunotherapy and overcoming heterogeneity-mediated resistance.

#### 2.1.3 Tumor cells themselves have natural resistance mechanisms

Beyond the well-characterized phenomenon of antigen escape, DLBCL tumor cells deploy a repertoire of potent intrinsic resistance programs that directly counteract CAR-T cell-mediated killing. These mechanisms primarily revolve around two core strategies: enhancing their own survival capacity and actively disrupting cytotoxic signaling transduction. This coordinated defense constitutes a robust secondary line of resistance, operating even after the primary barrier of antigen loss has been breached.

##### 2.1.3.1 Activation of inhibitory immune checkpoints

Beyond passive evasion through antigen loss, tumor cells actively upregulate a repertoire of inhibitory immune checkpoint molecules on their surface, delivering potent “off” signals to infiltrating CAR-T cells within the tumor microenvironment (TME) to directly suppress their immune efficacy. The PD-1/PD-L1 axis plays a central role in this process. Upon infiltration and release of interferon-gamma (IFN-γ) by CAR-T cells, DLBCL tumor cells reciprocally upregulate PD-L1 expression (41). The engagement of PD-L1 with PD-1 on CAR-T cells initiates inhibitory signaling, directly impairing CAR-T cell proliferation and cytokine production while accelerating their functional exhaustion (42, 43). This mechanism is particularly critical in subtypes like primary mediastinal large B-cell lymphoma (PMBCL) and T-cell/histiocyte-rich large B-cell lymphoma (THRLBCL), which frequently harbor amplifications of the PD-L1/PD-L2 genomic locus, resulting in constitutively high baseline expression (44, 45).

Concurrently, other inhibitory receptors establish additional barriers within the immunosuppressive DLBCL microenvironment. CTLA-4 represents another key inhibitory receptor, whose expression on CAR-T cells can be upregulated by factors in the TME, including regulatory T cells (Tregs) and their secretion of transforming growth factor-beta (TGF-β) (46). By outcompeting CD28 for binding to the costimulatory ligands CD80/CD86 at the immune synapse, CTLA-4 effectively hijacks costimulatory signals. This not only prevents full CAR-T cell activation but may also induce the expression of other inhibitory receptors like LAG-3, creating a cascade of negative regulation (47, 48). With prolonged antigen exposure, the TIM-3/Galectin-9 pathway emerges as a dominant driver of terminal CAR-T cell exhaustion. Binding of Galectin-9 to TIM-3 triggers disruptions in intracellular calcium homeostasis and progressive mitochondrial dysfunction in CAR-T cells, forcing a metabolic shift from efficient oxidative phosphorylation to less efficient glycolysis. This metabolic crisis directly cripples their capacity to synthesize and secrete cytotoxic mediators such as granzyme B (49, 50) (**Figure 1F**).

Critically, these inhibitory pathways do not operate in isolation but are intricately interconnected and synergistically amplified within the DLBCL TME, forming a resilient inhibitory network resistant to single-target interventions. Single-cell transcriptomic studies reveal that TIM-3-high CAR-T cell subsets are typically characterized by silenced memory-associated genes and sustained activation of exhaustion-associated transcription factors, marking a state of irreversible functional decline. This exhausted state can become epigenetically fixed (50); for instance, persistent antigen stimulation and PD-1 signaling can induce stable alterations in DNA methylation patterns within CAR-T cells, locking exhaustion-related genes in a highly accessible state even in the absence of antigen, thereby chronically impairing CAR-T cell function (43). This multi-layered, multi-target inhibitory network underscores the strategic necessity of combinatory blockade of multiple immune checkpoints (e.g., simultaneous targeting of PD-1 with LAG-3 or TIM-3) and provides a rationale for genetically engineering armored CAR-T cells—for example, by knocking out multiple inhibitory receptors—to create more resistant effector populations.

##### 2.1.3.2 Enhanced anti-apoptotic and pro-survival pathways

Furthermore, CAR-T cells primarily induce tumor cell apoptosis through the perforin-granzyme pathway and death receptor signaling. DLBCL tumor cells can resist this process by fortifying their intrinsic anti-apoptotic capabilities. Sustained overexpression of BCL-2 family proteins is highly prevalent in DLBCL; these proteins effectively block the mitochondrial apoptotic pathway by competitively binding and inhibiting the activity of pro-apoptotic effectors BAX/BAK (51). When this mechanism synergizes with frequently occurring TP53 loss-of-function mutations, tumor cells can even resist the extrinsic apoptosis pathway mediated by death receptors, thereby developing broad-spectrum resistance to the dual apoptotic signals triggered by CAR-T cells (52) (**Figure 1E**). Beyond apoptosis evasion, stress-induced activation of the autophagy pathway also provides critical protection for tumor cells. Under the metabolic pressure imposed by CAR-T cells, the expression of key autophagy regulators is systematically upregulated in tumor cells. This adaptive response not only clears damaged organelles to maintain cellular homeostasis but also directly suppresses the execution of apoptotic signaling by interfering with the activation of key proteins such as caspase-8 (53).

##### 2.1.3.3 Disruption of the immunological synapse

Moreover, effective cytotoxicity is critically dependent on the formation of a stable “immunological synapse” between the CAR-T cell and the tumor cell. DLBCL tumor cells can subvert this attack by disrupting this pivotal structure. CD58 (also known as LFA-3), a costimulatory molecule expressed on antigen-presenting cells and some tumor cells, binds to CD2 on CAR-T cells and is essential for stabilizing the immunological synapse and providing costimulatory signals. In DLBCL, loss of CD58 expression or functional mutations compromises immune synapse stability, resulting in impaired polarized release of cytotoxic granules and significantly reduced secretion efficiency of key effector molecules, ultimately drastically diminishing the clearance efficacy of CAR-T cells (54).

This observation underscores that the intrinsic resistance mechanisms of tumor cells often do not operate in isolation. For instance, a tumor cell exhibiting concurrent high expression of BCL-2 and PD-L1 would demonstrate profoundly robust resistance. The establishment of this multi-layered defense system strongly advocates for mechanism-based combination therapies. Preclinical studies have confirmed that combining CAR-T therapy with specific inhibitors of the BCL-2 family (e.g., venetoclax) can effectively lower the apoptosis threshold of tumor cells, demonstrating synergistic potential in eradicating tumors (55). Consequently, pre-therapeutic assessment of the tumor’s intrinsic resistance profile is crucial for selecting the most effective combination regimens.

### 2.2 Problems with the structure or function of CAR-T cells

#### 2.2.1 Structural design limitations

Beyond resistance mechanisms originating from tumor cells, the structural integrity of the CAR-T cell itself constitutes the molecular foundation for its anti-tumor efficacy. Subtle deviations in its engineered design can precipitate cascading functional impairments. As a synthetically constructed receptor, the CAR’s modular architecture—encompassing the antigen-binding domain, hinge region, transmembrane domain, and costimulatory domains—requires an exquisitely balanced interplay between spatial conformation and signaling fidelity. Compromise in any constituent module invariably leads to a substantial reduction in therapeutic potency against DLBCL through systemic functional decay (56).

##### 2.2.1.1 Structural compromises in antigen recognition domains: dysfunctional structure-activity relationships

The precise optimization of the antigen recognition domain presents a primary challenge in CAR design. Functioning as the molecular key of CAR-T cells, structural fine-tuning of the single-chain variable fragment (scFv) directly governs the accuracy and potency of antigen targeting (57). For clinical applications in DLBCL, the binding affinity of the scFv for the CD19 antigen must be maintained within an optimal range. Studies indicate that when the dissociation constant (Kd) increases significantly, the clearance efficiency of CAR-T cells against tumor cells with low CD19 antigen density is markedly impaired (58). a phenomenon particularly prominent in DLBCL tumors characterized by heterogeneous antigen expression. Furthermore, the degree of scFv humanization critically influences treatment durability. Murine framework residues retained in suboptimally humanized constructs can be recognized by the host immune system, potentially eliciting anti-CAR immune responses (59–62). and leading to premature elimination of CAR-T cells. This has driven the development of fully humanized or highly humanized scFvs to extend the persistence of CAR-T cells *in vivo*.

##### 2.2.1.2 Structural dynamic defects in hinge and transmembrane domains​

The structural coupling between the hinge and transmembrane domains collectively underpins the stability and signaling fidelity of the CAR molecule. The length and composition of the hinge region directly influence the spatial flexibility of the antigen-binding domain, thereby impacting the efficiency of immunological synapse formation. For instance, while long hinge regions derived from immunoglobulins (e.g., IgG4) can enhance flexibility, their latent Fcγ receptor (FcγR)-binding motifs may trigger antigen-independent, off-target activation of CAR-T cells, consequently accelerating their terminal differentiation and exhaustion (63, 64). The selection of the transmembrane domain (TMD) governs the assembly mode and stability of the CAR receptor. Using the CD3ζ-TMD as an example, it promotes CAR homodimerization and facilitates physical co-clustering with endogenous TCR signaling complexes. Although this tight aggregation can amplify initial activation signals, it is counterbalanced by accelerated CAR degradation and an elevated risk of activation-induced cell death (AICD), ultimately constraining the *in vivo* expansion and persistence of CAR-T cells (65). Further investigations reveal that electrostatic complementarity between the hinge and transmembrane domains is critical for preventing non-physiological oligomerization of the CAR molecule (66). The functional cooperativity between these two domains is essential for maintaining normal T cell effector output; however, systematic research in this specific area remains relatively limited.

##### 2.2.1.3 Spatiotemporal coordination of costimulatory signals is dysregulated

The structural integration of costimulatory signals represents another core determinant governing CAR-T cell fate and functionality, with domain selection directly steering divergent metabolic programs and differentiation trajectories. In the clinical practice of DLBCL, costimulatory domains derived from CD28 and 4-1BB exhibit distinct biological properties. CD28 costimulation potently activates the PI3K-AKT pathway, driving a metabolic shift toward glycolysis in T cells. While this can rapidly generate potent effector function, prolonged antigen exposure under this signaling paradigm predisposes cells to metabolic exhaustion and functional decline (67). potentially contributing to the higher rates of secondary resistance observed with some CD28-based CAR-T products in DLBCL treatment (68). In striking contrast, 4-1BB signaling, transduced via the TRAF2-NF-κB axis, preferentially promotes mitochondrial biogenesis and fatty acid oxidation (69–71). This metabolic profile is more conducive to the maintenance of a memory phenotype and long-term persistence, demonstrating a unique advantage in DLBCL therapy which often requires sustained tumor control (72, 73).Notably, CD4+ T cell subsets, characterized by relatively limited mitochondrial reserves, exhibit heightened sensitivity to the metabolic stress induced by CD28 signaling (74). The 4-1BB pathway can effectively rescue fatty acid oxidation flux in these cells by upregulating the expression of carnitine palmitoyltransferase CPT1A (75). This provides a metabolic rationale for the differential impact of costimulatory domains on T cell subset functionality and informs the design of more balanced CAR products.

#### 2.2.2 Abnormal function of CAR-T cells

During the treatment of DLBCL, the functional exhaustion of CAR-T cells *in vivo* represents a fundamental limitation to their long-term efficacy. This pathological state is intrinsically driven by persistent antigen exposure, which triggers a systemic dysregulation of intrinsic cellular programs. It is characterized by metabolic dysregulation, epigenetic imbalance, and an irreversible commitment toward a terminal effector state, ultimately culminating in the comprehensive decline of effector functions.

##### 2.2.2.1 Signal pathway dysregulation and metabolic imbalance

**Figure 2:**
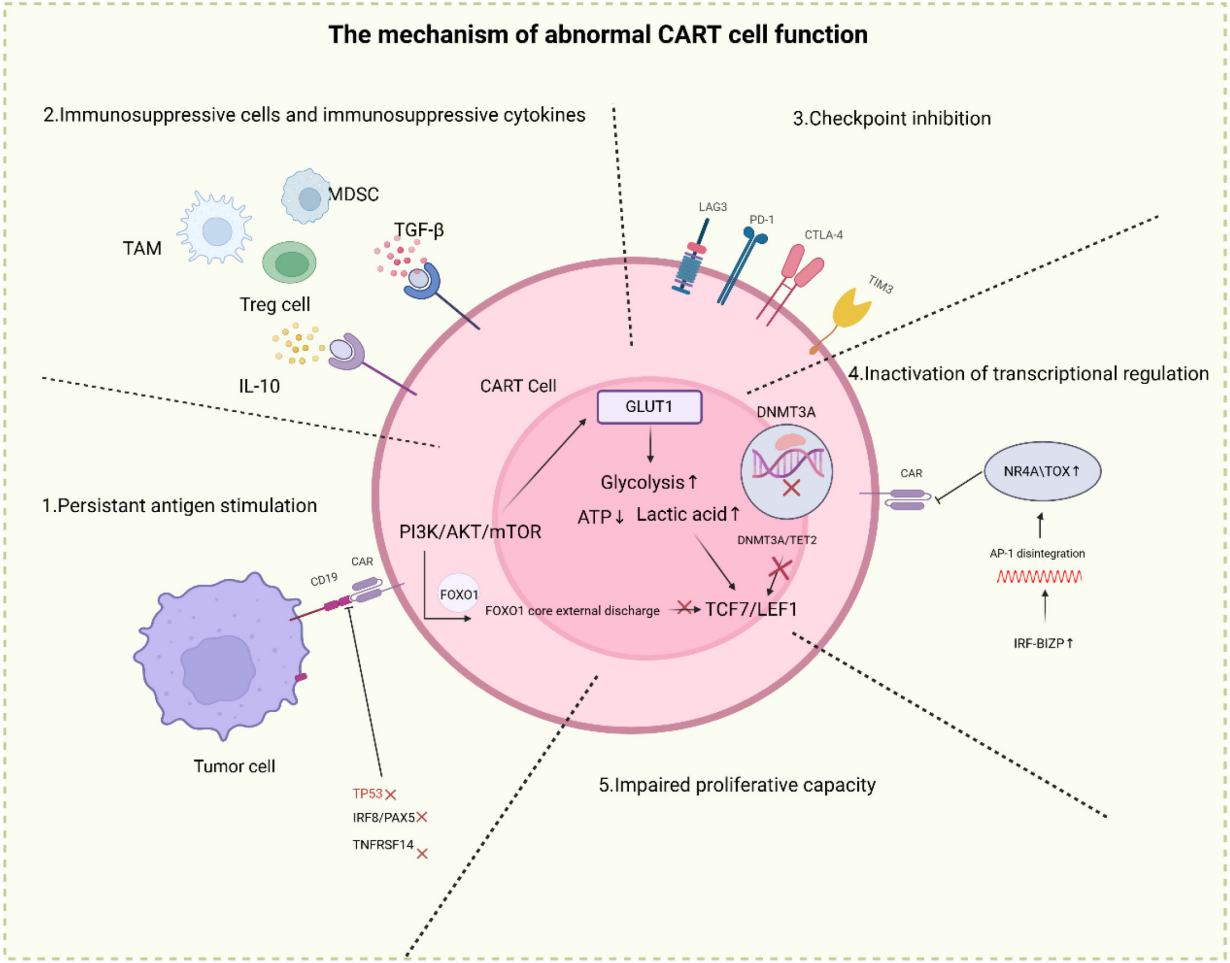
The mechanism of abnormal CART function. This schematic illustrates the key pathways driving CAR-T cell dysfunction within the tumor microenvironment: (1) Persistent antigen stimulation: Sustained engagement with CD19-expressing tumor cells impairs CAR-T signaling (e.g., downregulation of TPSX, INF6/PAX5, TNFSF14); (2) Immunosuppressive cues: Tumor-associated macrophages (TAM), myeloid-derived suppressor cells (MDSC), regulatory T cells (Treg), and soluble factors (transforming growth factor-β [TGF-β], interleukin-10 [IL-10]) directly suppress CAR-T activity; (3) Checkpoint-mediated inhibition: Upregulation of inhibitory checkpoint molecules (programmed cell death protein 1 [PD-1], cytotoxic T-lymphocyte-associated protein 4 [CTLA-4], lymphocyte-activation gene 3 [LAG3]) constrains CAR-T effector function; (4) Transcriptional/epigenetic dysregulation: Impaired activity of regulators (e.g., DNA methyltransferase 3A [DNMT3A], nuclear receptor subfamily 4 group A/thymocyte selection-associated high mobility group box [NR4A/TOX1], activator protein 1 [AP-1], interferon regulatory factor 8/B lymphocyte-induced maturation protein 1 [IRF8-BLZ1]) disrupts CAR-T fate programming; (5) Metabolic and proliferative impairment: Deregulated glycolysis (via glucose transporter 1 [GLUT1]) and altered signaling (e.g., nuclear export of forkhead box O1 [FOXO1], downregulation of T cell factor 7/lymphoid enhancer-binding factor 1 [TCF7/LEF1]) compromise CAR-T survival and expansion.

In the pathological context of DLBCL, persistent antigen exposure serves as the initiating factor driving T cell exhaustion, primarily by triggering sustained signaling imbalance and erroneous metabolic reprogramming within CAR-T cells. Repeated stimulation of the CAR structure within the tumor microenvironment leads to aberrant, persistent activation of the PI3K/AKT/mTOR signaling axis. Pathological hyperactivation of this pathway forces a metabolic shift from efficient oxidative phosphorylation toward a glycolysis-dominant energy supply (76, 77). While this metabolic adaptation ostensibly supports rapid effector demands, it ultimately results in diminished ATP synthesis efficiency and accumulation of metabolic waste, precipitating a cellular energy crisis. More critically, this metabolic imbalance is tightly coupled to T cell differentiation fate: activated AKT mediates phosphorylation and subsequent nuclear exclusion of the transcription factor FOXO1, directly suppressing the transcription of genes associated with T cell memory formation and self-renewal, such as TCF7 and LEF1 (78) (**Figure 2**). Concurrently, aberrant accumulation of acetyl-CoA from heightened glycolysis acts as a key epigenetic modulator, promoting acetylation of histone H3 at lysine 27 (H3K27ac) and thereby forcibly opening chromatin loci associated with terminal effector phenotypes (79, 80) The synergistic action of this signaling dysregulation and metabolic remodeling collectively propels CAR-T cells toward an irreversible commitment to a short-lived terminal effector phenotype, accompanied by a marked erosion of their self-renewal capacity and long-term persistence. Preclinical evidence confirms that pharmacological inhibition of this cascade effectively preserves FOXO1 nuclear localization and significantly augments the frequency of central memory T cells (81)​​. This not only underscores the central role of signaling pathway dysregulation in CAR-T cell exhaustion but also provides a mechanistic rationale for metabolic intervention strategies aimed at reversing treatment resistance in DLBCL.

##### 2.2.2.2 Epigenetic reprogramming and exhaustion commitment

Beyond acute signaling dysregulation, persistent antigen exposure induces profound epigenetic reprogramming that underlies the irreversible functional exhaustion of CAR-T cells. This process hinges on the disruption of DNA methylation homeostasis, wherein the dioxygenase TET2 and the methyltransferase DNMT3A play pivotal roles.

Under chronic stimulation within the DLBCL microenvironment, pathological activation of DNMT3A imposes a hypermethylation barrier at the regulatory regions of stemness-associated genes such as TCF7 and LEF1. This functions as “epigenetic gatekeeping,” hindering CAR-T cell differentiation toward memory subsets and consequently restricting their long-term antitumor potential (82). Conversely, loss-of-function of TET2, a key regulator of active DNA demethylation, elicits a more complex phenotypic outcome: promoter hypermethylation leads to suppressed expression of effector/co-stimulatory genes like IFNG and NOTCH2, while the expression of cytotoxic molecules including granzyme B and perforin may be paradoxically enhanced. This creates a dissociation between cytotoxic potential and memory traits, revealing TET2’s dual regulatory role in coordinating effector function and long-term persistence in CAR-T cells (83).

Beyond DNA methylation, aberrations in histone modification and their recognition systems are deeply implicated. Bromodomain and extra-terminal (BET) family proteins (e.g., BRD4) act as critical chromatin “readers.” They recognize histone acetylation marks and recruit various modifiers, including TET2, collectively maintaining exhaustion-associated gene clusters in an aberrantly open and hyperactive chromatin state. This BET-mediated epigenetic “platform” stabilizes the exhaustion program (**Figure 2**). Consequently, specific inhibition of BET proteins not only disrupts their interaction with partners like TET2 but also significantly reduces the expression of exhaustion markers such as PD-1 and TIM-3, identifying a novel target for epigenetically “resetting” CAR-T cell function (84).

##### 2.2.2.3 Transcriptional network inactivation​​

Furthermore, the collapse of core transcriptional networks underlies the profound functional exhaustion of CAR-T cells, with structural disintegration of the AP-1/JUN complex proving particularly consequential. Under physiological T-cell activation, Fos/Jun proteins form heterodimers to regulate genes governing proliferation and survival. However, under the persistent antigenic pressure of the DLBCL microenvironment, aberrant activation of the IRF-bZIP complex creates a dynamic imbalance that disrupts AP-1 DNA-binding capacity. This disruption precipitates pathological accumulation of the exhaustion-associated transcriptional repressors TOX and the orphan nuclear receptor NR4A, establishing a self-perpetuating cycle of sustained inhibitory receptor expression (85). Concurrently, the function of the BATF-IRF4 transcriptional complex undergoes a critical shift, transitioning from its role in promoting effector molecule expression to actively driving exhaustion programming (86, 87). Furthermore, transcriptional regulatory systems such as ID3/SOX4, which normally serve as antigen-response brakes, are paradoxically transformed into accelerators of the exhaustion program under chronic stimulation (**Figure 2**) (88). Collectively, these aberrations in transcriptional control map the molecular circuitry of CAR-T cell exhaustion in DLBCL, suggesting that hierarchical regulation of these transcriptional hubs may represent a breakthrough avenue for next-generation CAR-T engineering.

##### 2.2.2.4 Host genetic background as a critical determinant in CAR-T efficacy​​

Beyond defects in effector molecules, the host genetic background acts as an “invisible director” in shaping the therapeutic outcomes of CAR-T cell therapy. Specific genetic variations in the patient can disrupt immune recognition, signal transduction, and intrinsic properties of tumor cells, thereby establishing a pre-existing framework that influences efficacy and resistance from the outset of treatment.

Firstly, at the level of antigen recognition and immune synapse formation, the host genetic background interferes with CAR-T cell cognition by altering the “identity markers” of tumor cells. Loss-of-function mutations in key B-cell lineage transcription factors, such as IRF8 and PAX5, directly lead to significant downregulation of Major Histocompatibility Complex Class II (MHC-II) molecule expression on tumor cells. This “immune recognition impairment” creates a dual vulnerability: it not only diminishes the efficiency of CAR-T cell recognition of the CD19 target antigen but, more critically, also hampers the cross-presentation and recognition of tumor neoantigens by the host’s endogenous T cells, thereby undermining the overall antitumor immune response upon which the therapy relies (89). Secondly, in the establishment of immunoregulatory signals, structural variations in the host genome provide tumor cells with innate tools for immune evasion. A prime example is the aberrant amplification of the CD274/PD-L1 gene locus, which is particularly common in specific subtypes like primary mediastinal large B-cell lymphoma (PMBCL). This alteration, originating from the host tumor genome, results in constitutive overexpression of PD-L1 on the tumor cell surface. This means CAR-T cells confront a pre-established, potent inhibitory barrier from the moment of infiltration, making their activation an uphill battle (44).​​ Similarly, functional aberrations in TNFRSF14 (HVEM) on tumor cells can directly inhibit activation signaling within CAR-T cells by recruiting phosphatases such as SHP-1/SHP-2, further exacerbating their functional exhaustion (90). These findings underscore the importance of pre-treatment genotyping. For instance, in patients identified with PD-L1 gene amplification, a strategic consideration of combining CAR-T therapy with immune checkpoint inhibitors from the initiation of treatment might be fundamentally more rational.

Furthermore, genomic instability in key prognostic genes fundamentally shapes the resilient nature of tumor cells, making them notoriously difficult to eradicate. Inactivation mutations in TP53, the “guardian of the genome,” occurring in the host’s tumor cells, not only accelerate genomic instability and heterogeneity in DLBCL but also promote replicative immortality of tumor cells through mechanisms such as telomerase dysregulation, thereby conferring enhanced evolutionary and adaptive capacity (91). On the other hand, loss-of-function of the transmembrane protein TMEM30A compromises phospholipid flippase activity, altering the externalization of phosphatidylserine on the cell membrane. This, in turn, amplifies the “don’t eat me” signaling mediated by the CD47-SIRPα axis, further reinforcing the tumor cell’s ability to evade innate immunity (92, 93) (**Figure 2**). These mechanisms are conserved and exert profound effects, strongly suggesting that comprehensive genomic profiling of patients prior to CAR-T therapy is paramount. Identifying these high-risk genetic features not only enables more accurate prediction of treatment efficacy but also provides a rationale for implementing personalized, preemptive combination strategies.

### 2.3 Microenvironment issues

The tumor microenvironment (TME) is the main battlefield for CAR-T cells to achieve sustained anti-tumor activity, and its complex inhibitory network induces drug resistance through multiple mechanisms.

#### 2.3.1 Immune-suppressive cell populations

Within the landscape of tumor immunotherapy, concerted actions of immune-suppressive cells in the DLBCL tumor microenvironment (TME) critically undermine CAR-T cell efficacy. This sophisticated cellular alliance—primarily comprising regulatory T cells (Tregs), myeloid-derived suppressor cells (MDSCs), and tumor-associated macrophages (TAMs)—collectively attenuates CAR-T cell anti-tumor activity through multifaceted mechanisms, thereby establishing a robust immune evasion network.

Tregs serve as pivotal immunosuppressive sentinels within the TME. They directly impair CAR-T cell proliferation and cytotoxicity through secretion of immunosuppressive cytokines such as transforming growth factor-beta (TGF-β) and interleukin-10 (IL-10). Concurrently, Tregs exploit their surface overexpression of CD25 (IL-2Rα chain) to competitively sequester IL-2 within the TME. This molecular deprivation starves CAR-T cells of essential cytokine signals, substantially curtailing their *in vivo* persistence and therapeutic potency (94, 95).

MDSCs mount a dual blockade against CAR-T cells at metabolic and signaling levels. The overexpression of arginase-1 (ARG1) in MDSCs catabolizes arginine, depleting this crucial amino acid in the TME (96, 97). This arginine scarcity destabilizes the CD3ζ chain of the T-cell receptor, compromising CAR-T activation (98). Furthermore, MDSCs aberrantly upregulate programmed death-ligand 1 (PD-L1). Subsequent binding to PD-1 receptors on CAR-T cells chronically disrupts CD28 costimulatory signaling efficiency, further crippling CAR-T functionality (99).

TAMs, particularly the M2-polarized subset, exhibit even more diverse suppressive functions within the DLBCL immunosuppressive network. They secrete copious IL-10, TGF-β, and chemokines like CCL22, thereby recruiting additional Tregs into tumor cores to amplify immunosuppression through a positive feedback loop (100) (**Figure 2**). Crucially, the CD47 molecule on tumor cells engages SIRPα receptors on TAMs, transmitting an intrinsic “don’t-eat-me” inhibitory signal. This interaction prompts TAMs to misidentify CAR-T cells as non-targets, leading to Fcγ receptor-mediated phagocytosis and physical eradication of CAR-T effectors—severely diminishing their operational capacity within tumors (101–103). This direct physical clearance mechanism poses a fundamental threat to CAR-T therapy efficacy, extending beyond mere functional suppression.

In summary, immunosuppressive populations within the TME do not function in isolation but establish a coordinated defense system. Understanding the dominance of specific cell types within particular DLBCL subtypes—for instance, focusing on Tregs in THRLBCL, or on MDSCs and TAMs in subtypes rich in myeloid infiltration—provides crucial guidance for developing targeted combination therapies aimed at dismantling this suppressive network.

#### 2.3.2 Metabolic dysregulation and nutrient deprivation​​

The tumor microenvironment (TME) establishes a metabolically hostile milieu that systemically compromises CAR-T cell function through multifaceted mechanisms, including nutrient deprivation, accumulation of toxic metabolites, and generation of inhibitory molecules.

**Figure 3:**
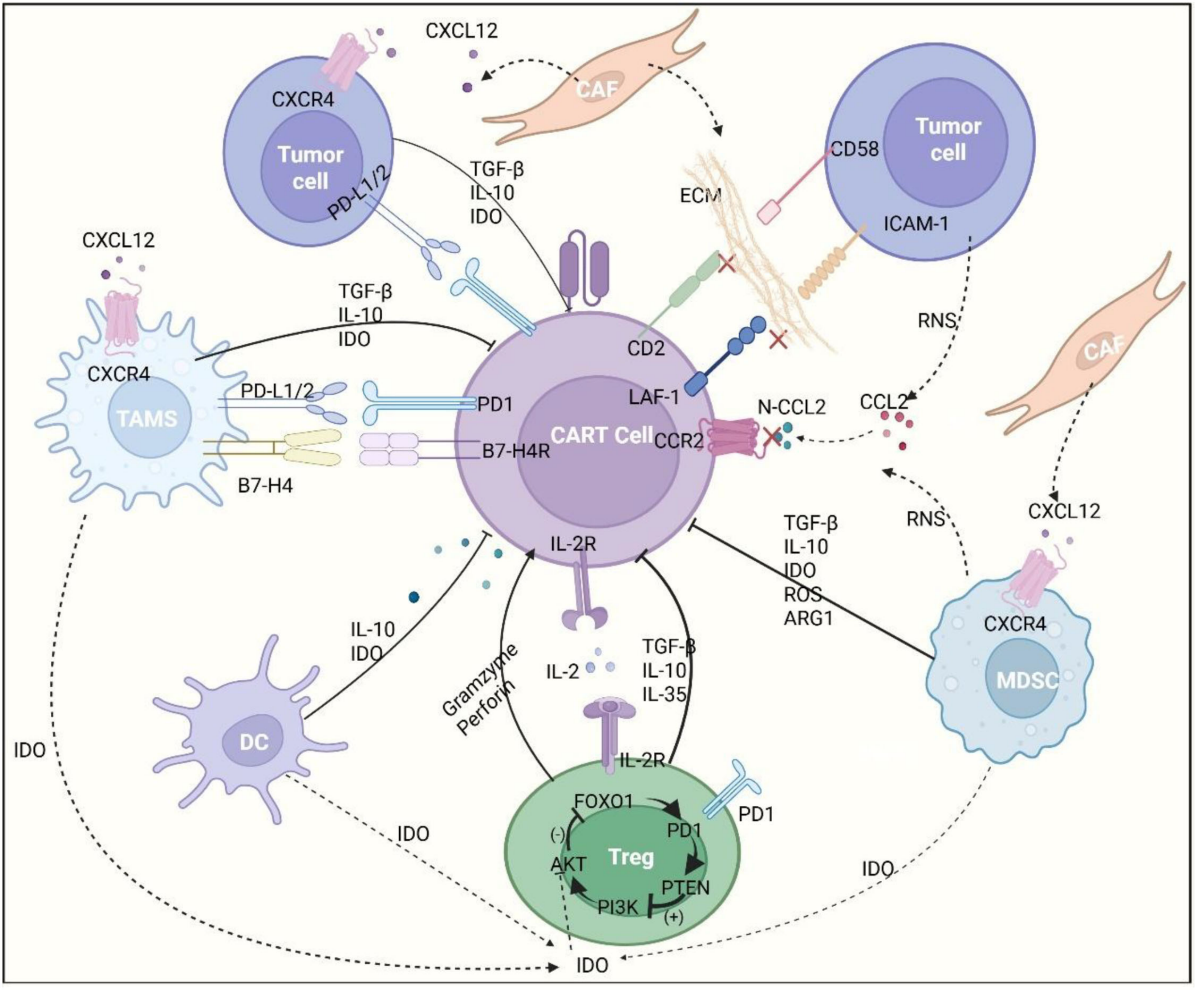
Schematic illustration of CAR-T cell dysfunction mediated by multiple cellular components in the tumor microenvironment (TME.). In the TME, heterogeneous cellular subsets—including tumor cells, tumor-associated macrophages (TAMs), cancer-associated fibroblasts (CAFs), myeloid-derived suppressor cells (MDSCs), dendritic cells (DCs), and regulatory T cells (Tregs)—orchestrate a suppressive network that impairs CAR-T cell function. Key inhibitory mechanisms include: (1) chemokine-mediated recruitment/retention (e.g., CXCL12 secreted by CAFs/tumor cells engaging CXCR4 on CAR-T cells, TAMs, and MDSCs); (2) secretion of immunosuppressive cytokines and enzymes (e.g., TGF-β, IL-10, indoleamine 2,3-dioxygenase (IDO) from tumor cells, TAMs, DCs, and MDSCs; IL-35 from Tregs); (3) engagement of inhibitory receptor-ligand axes (e.g., PD-L1/PD-1, B7-H4/B7-H4R between TME cells and CAR-T cells); (4) physical barriers from extracellular matrix (ECM) (e.g., disrupted CD2/LFA-1-ICAM-1 interactions between CAR-T and tumor cells); (5) oxidative/nitrosative stress (reactive oxygen species (ROS)/reactive nitrogen species (RNS)) and metabolic dysregulation (e.g., arginase 1 (ARG1) from MDSCs); and (6) modified chemokine signaling (e.g., nitrated CCL2 (N-CCL2) impairing CAR-T cell migration via CCR2). While CAR-T cells exert cytotoxicity via granzyme and perforin, Tregs further reinforce suppression through intracellular signaling regulation (e.g., PI3K/AKT-mediated inhibition of FOXO1, with PTEN counteracting PI3K activity). Collectively, these TME-driven mechanisms synergistically attenuate CAR-T cell activation, proliferation, and antitumor efficacy.

Regarding nutrient competition, tumor cells overexpress glucose transporter GLUT1 to monopolize glucose supplies within the TME (104, 105). This metabolic hijacking forces glycolysis-dependent CAR-T cells into an energy crisis. The resulting glucose scarcity not only depletes ATP synthesis but also crucially depletes the energy required for microtubule polarization during immune synapse assembly, directly impairing tumor recognition capabilities (106). Concurrently, indoleamine 2,3-dioxygenase (IDO)-mediated tryptophan catabolism constitutes another layer of nutrient stress. IDO catalysis depletes tryptophan while generating kynurenine metabolites. Tryptophan scarcity both activates GCN2 kinase to phosphorylate eIF2α—triggering the integrated stress response and blocking effector protein translation (107) —and suppresses the mTORC1 signaling hub, disabling metabolic adaptability in CAR-T cells (108) (**Figure 3**).

When glucose scarcity and hypoxia form a mutually reinforcing cycle, the accumulation of aberrant metabolites further exacerbates the immunosuppressive state. For instance, in DLBCL subtypes with a strong glycolytic preference, substantial lactate production contributes to microenvironmental acidification and may indirectly promote apoptosis sensitivity in CAR-T cells, potentially through influencing epigenetic modification patterns and mitochondrial function (109)​. Notably, this metabolic dysregulation synergizes with specific epigenetic programs—such as EZH2-mediated deposition of H3K27me3—collectively enforcing persistent silencing of memory-associated transcription factors (e.g., TCF7, LEF1) while stabilizing exhaustion drivers (e.g., BATF, IRF4) in T cells. This dynamic ultimately promotes the establishment of an antigen-independent “exhausted memory” phenotype, thereby perpetuating a long-term immunosuppressive state within the microenvironment (86). Furthermore, the ectoenzyme axis of CD39/CD73 on tumor cells converts extracellular ATP into adenosine, creating elevated adenosine concentrations in the TME. Binding of adenosine to the A2a receptor (A2aR) on CAR-T cells initiates cAMP-PKA signaling cascades, which subsequently impair T-cell receptor signal transduction, significantly compromising CAR-T cell functionality (110). Targeting this pathway, either with A2aR antagonists or through genetic deletion of A2aR in CAR-T cells, has emerged as a viable strategy to restore their effector function.

In conclusion, the DLBCL TME, through intricate metabolic rewiring, constructs a profoundly adverse “metabolic quagmire” for CAR-T cells. Future combination strategies must incorporate metabolic interventions to neutralize this suppressive landscape and create a supportive metabolic environment conducive to CAR-T cell function.

#### 2.3.3 Physical barriers and the stromal compartment

Within the progression of DLBCL, the tumor microenvironment (TME) not only suppresses immune cell function biochemically but also constructs complex physical barriers and stromal networks that pose significant mechanical impediments to CAR-T cell infiltration, migration, and target engagement, effectively transforming the tumor core into an “immunologically privileged niche” refractory to CAR-T penetration.

The remodeling of the extracellular matrix (ECM) constitutes the primary physical barrier to tumor immune evasion. Type I collagen significantly impedes CAR-T cell motility through integrin (e.g., α2β1)-mediated focal adhesion kinase (FAK) phosphorylation, which enhances cell-matrix adhesion forces. This interaction immobilizes CAR-T cells within high-stiffness ECM niches (111, 112), drastically reducing their migratory capacity. Concurrently, excessive accumulation of proteoglycans like hyaluronic acid (HA) not only directly occupies physical space and hinders T cell movement via its bulky hydrophilic molecular chains but also fosters the development of PD-L1-expressing macrophages. This establishes a localized microenvironment possessing both physical and immunosuppressive properties, thereby multilaterally attenuating CAR-T-mediated antitumor responses (113). Furthermore, the ECM is enriched with heparan sulfate proteoglycans (HSPGs), posing another formidable challenge. CAR-T cells inherently lack heparanase, the critical enzyme required for cleaving HSPGs, rendering them largely incapable of navigating this reticular meshwork and severely compromising their infiltration efficiency into tumor parenchyma (114). Compounding this physical obstruction, the ECM often exhibits a scarcity or downregulation of chemokines essential for CAR-T migration (e.g., CXCL9, CXCL10). This dysregulation compromises chemokine receptor-mediated recruitment, increasing CAR-T retention in non-tumor regions and significantly diminishing tumor-targeting efficiency (115).

Tumor-associated stromal cells play a synergistic role in barrier formation. Cancer-associated fibroblasts (CAFs) secrete basement membrane components such as laminin-511, which engages integrins on tumor cells to form mechanically robust, protective cellular encapsulation structures (116). Moreover, these cells can establish direct intercellular communication with tumor cells via connexin 43 (Cx43) and recruit CXCR4^+^ immunosuppressive cells while simultaneously excluding effector T cells from the tumor core (117, 118), thereby actively shaping a cellular distribution landscape unfavorable to CAR-T cells at the spatial level. This interplay creates a vicious cycle where physical stromal obstruction and chemical suppression by immune-inhibitory cells mutually reinforce each other, establishing a profoundly resilient defense network. Consequently, developing combination strategies aimed at enhancing CAR-T cell infiltration and distribution—for instance, co-administering agents that target CAFs, degrade hyaluronic acid, or disrupt collagen—holds promise for “softening” the tumor stroma and carving out accessible pathways for CAR-T cells to reach their battlefield.

#### 2.3.4 Inhibitory soluble factor networks

Beyond complex physical barriers and stromal components, the DLBCL tumor microenvironment harbors diverse soluble inhibitory factors that establish sophisticated immune escape systems at the molecular level, significantly attenuating CAR-T cell anti-tumor efficacy through multidimensional synergistic actions.

Transforming growth factor-beta (TGF-β) occupies a central position within this inhibitory network. Multiple studies demonstrate that TGF-β is collectively secreted by various sources, including tumor cells, regulatory T cells (Tregs), and cancer-associated fibroblasts (CAFs). This cytokine directly impedes CAR-T cell proliferation, disrupts effector differentiation, and markedly downregulates critical cytotoxic mediators including granzyme B—thereby multilaterally compromising CAR-T cell cytotoxicity (119, 120). Importantly, TGF-β-mediated suppression establishes a permissive foundation for synergistic inhibition by other factors. Interleukin-10 (IL-10), abundantly produced by tumor-associated macrophages (TAMs) and myeloid-derived suppressor cells (MDSCs), downregulates the surface expression of co-stimulatory molecules such as CD28 on CAR-T cells, thereby obstructing their activation signaling pathways (121). This functional impairment significantly compounds the proliferative suppression imposed by TGF-β. Preclinical studies indicate that blocking IL-10 signaling significantly enhances antitumor immunity in models of colorectal cancer liver metastases, providing a proof-of-concept for exploring similar combination strategies in DLBCL (122). Prostaglandin E2 (PGE2) represents another key participant in TME suppression. Secreted by tumor cells and MDSCs, PGE2 concurrently induces PD-L1 upregulation on CAR-T cells (123). Such dual actions not only directly compromise CAR-T anti-tumor activity but also amplify functional exhaustion through enhanced immune checkpoint inhibition, thereby linking metabolic inflammation with the immune checkpoint axis.

These inhibitory soluble factors do not operate in isolation but form spatially and functionally integrated networks within the TME. Their intricate interactions generate cascaded amplification of suppression via synergistic effects, collectively driving substantial CAR-T functional impairment and tumor immune escape (**Figure 3**). Therefore, future strategies to overcome this barrier may necessitate not only targeting individual factors but also considering combination regimens capable of simultaneously intervening at multiple key nodes, or employing engineering approaches to endow CAR-T cells with broad-spectrum resistance to such soluble inhibitory signals.

#### 2.3.5 Iatrogenic effects and copper dysregulation

Therapeutic refractoriness within the tumor microenvironment constitutes a complex biological phenomenon extending beyond inherent cancer cell properties. It manifests through two interdependent mechanisms: iatrogenic self-sabotage resulting from clinical interventions, and immune evasion potentiated by ion homeostasis dysregulation. These dual axes establish complementary resistance frameworks that collectively represent critical bottlenecks limiting CAR-T cell therapeutic efficacy.

Regarding iatrogenic self-sabotage from clinical interventions, management strategies for cytotoxic and immune-related adverse events may exert dual effects on CAR-T cell functionality. First, antibiotic exposure has been associated with compromised efficacy, primarily mediated through disruption of the gut microbiome. Studies demonstrate that administration of high-risk antibiotics (e.g., meropenem, cefepime) prior to CD19 CAR-T cell therapy significantly reduces treatment response rates in DLBCL patients, with this correlation being particularly pronounced in the lymphoma patient subgroup. A plausible explanation is that antibiotic-induced gut dysbiosis depletes specific probiotic populations capable of positively modulating systemic immunity and T-cell function through mechanisms such as molecular mimicry or microbial metabolites (e.g., short-chain fatty acids), thereby indirectly impairing CAR-T cell expansion and persistence (124). This observation suggests that judicious assessment of antibiotic necessity in clinical practice, alongside exploring microbiome-modulating strategies like probiotics or fecal microbiota transplantation as adjunct therapies, may hold potential value. Furthermore, glucocorticoid management for cytokine release syndrome (CRS) and immune effector cell-associated neurotoxicity syndrome (ICANS) requires careful evaluation. While early prophylactic use of corticosteroids for severe CRS does not appear to compromise the efficacy of CD19 CAR-T cells, prolonged or high-dose glucocorticoid (GC) exposure significantly upregulates exhaustion markers such as PD-1 and TIM-3 on CAR-T cells (125). More critically, GCs concurrently suppress both CD28 costimulatory signaling and IL-2 secretion, thereby impairing the maintenance and functionality of central memory T-cell (TCM) subsets. This therapy-related immunosuppression may synergize with intrinsic DLBCL immune evasion mechanisms, further constraining the long-term antitumor activity of CAR-T cells (126).

Beyond the potential drawbacks of clinical interventions, dysregulation of ion homeostasis within the tumor microenvironment is emerging as a novel mechanism of resistance. Exemplified by copper ion (Cu²^+^) imbalance, metabolic perturbations of this metal in the DLBCL microenvironment can influence treatment outcomes through multiple pathways. Investigations reveal that excessive Cu²^+^ impairs the normal function of the zinc finger domains in the p53 protein, consequently compromising DNA damage repair processes. Furthermore, elevated copper concentrations can promote PD-L1 expression—either by activating STAT3-dependent transcription or by suppressing ubiquitin-proteasome system (UPS)-mediated PD-L1 degradation—thereby facilitating cancer immune escape. In preclinical studies, combining the copper chelator tetrathiomolybdate with CD19 CAR-T therapy demonstrated a promising synergistic effect in DLBCL models. This strategy significantly augmented the population and cytotoxic efficiency of tumor-infiltrating CD8^+^ T cells, validating the critical importance of correcting ion homeostasis dysregulation for enhancing CAR-T efficacy (127) (**Table 1**).

**Table 1:** Multidimensional mechanisms of resistance to CAR-T cell therapy in diffuse large B-cell lymphoma (DLBCL).

Resistance mechanism	Research focus	Key findings	References
1. Tumor Cell-Intrinsic mechanisms			
Antigen escape	CD19 mutations, exon skipping, intron retention, promoter hypermethylation	30–40% of relapsed patients show CD19 loss; frameshift mutations and biallelic inactivation are common.	(2, 11–15)
Tumor heterogeneity & clonal evolution	Pre-existing CD19-negative subclones, APOBEC3-mediated mutagenesis, spatial heterogeneity	CD19-negative clones expand under CAR-T pressure; subclonal cooperation promotes immune escape.	(12, 34–39)
Intrinsic resistance programs	Upregulation of PD-L1, BCL-2 overexpression, CD58 loss, autophagy activation	Enhanced anti-apoptotic signaling and immune checkpoint expression impair CAR-T cytotoxicity.	(41–55)
2. CAR-T Cell-Related deficiencies			
Structural design limitations	scFv affinity, humanization, hinge/TM domain optimization, costimulatory signaling	Low-affinity scFv and non-humanized domains reduce efficacy and persistence; CD28 vs. 4-1BB impacts metabolism.	(56–75)
Functional exhaustion & epigenetic remodeling	PI3K/AKT/mTOR signaling, TET2/DNMT3A activity, BET proteins, AP-1/JUN complex	Chronic signaling leads to metabolic dysregulation, epigenetic exhaustion, and transcriptional collapse.	(76–89)
Host genetic background	IRF8/PAX5 mutations, PD-L1 amplification, TP53 loss, TMEM30A dysfunction	Alters antigen presentation, enhances immune evasion, and promotes genomic instability.	(89–93)
3. Tumor Microenvironment (TME)			
Immunosuppressive cells	Tregs, MDSCs, TAMs via cytokines, arginine depletion, PD-L1, CD47-SIRPα axis	Suppress CAR-T function through cytokine deprivation, metabolic interference, and phagocytosis.	(94–103)
Metabolic dysregulation	Glucose/tryptophan deprivation, lactate accumulation, adenosine via CD39/CD73	Nutrient competition and toxic metabolites impair CAR-T energy metabolism and synaptic function.	(104–110)
Physical barriers	Collagen/HA-rich ECM, CAFs, lack of chemokines (e.g., CXCL9/10)	Impede CAR-T infiltration and retention; create immune-excluded niches.	(111–118)
Soluble inhibitory factors	TGF-β, IL-10, PGE2, VEGF	Inhibit CAR-T proliferation, cytotoxicity, and costimulatory signaling.	(119–127)
Clinical & ion dysregulation	Antibiotics, glucocorticoids, copper imbalance	Microbiome disruption, exhaustion exacerbation, and PD-L1 upregulation via Cu²^+^.	(124–127)
4. Integrated & Subtype-Specific Resistance			
THRLBCL	PD-L1 overexpression, sparse tumor cells in T-cell-rich stroma	Physical and functional barriers lead to high primary resistance.	(45, 190)
PMBCL	9p24.1 amplification → PD-L1/PD-L2 and JAK2 overexpression	Dual resistance: immune checkpoint + metabolic competition.	(44, 191)
DHL/THL	BCL-2/MYC overexpression, immunosuppressive TME	Anti-apoptotic + microenvironmental suppression → broad therapy resistance.	(9, 51, 52)

This table summarizes key resistance mechanisms to CAR-T therapy in DLBCL, including antigen loss, tumor heterogeneity, T-cell dysfunction, and immunosuppressive microenvironment, which often interact to drive treatment failure.

CAR-T, Chimeric Antigen Receptor T-cell; DLBCL, Diffuse Large B-cell Lymphoma; TME, Tumor Microenvironment; scFv, single-chain variable fragment; Tregs, Regulatory T Cells; MDSCs, Myeloid-Derived Suppressor Cells; TAMs, Tumor-Associated Macrophages; ECM, Extracellular Matrix; CAFs, Cancer-Associated Fibroblasts; PD-1, Programmed Cell Death Protein 1; PD-L1, Programmed Death-Ligand 1; THRLBCL, T-cell/Histiocyte-Rich Large B-cell Lymphoma; PMBCL, Primary Mediastinal B-cell Lymphoma; DHL/THL, Double-Hit/Triple-Hit Lymphoma

## 3 Strategies to overcome diffuse large B-cell lymphoma resistance to CAR-T cell therapy

### 3.1 Multi-targeting strategies: counteracting antigen escape

The loss or downregulation of tumor antigens is a central mechanism of resistance in CAR-T therapy, particularly prominent in aggressive B-cell lymphomas, where it directly contributes to relapse following single-target CAR-T treatment. To address this clinical challenge, multi-targeting CAR-T strategies have been developed, with a core design logic focused on simultaneous targeting of multiple tumor-associated antigens. This approach broadens the recognition spectrum of T cells against tumor cells, thereby fundamentally reducing the risk of immune escape due to the loss of any single antigen and offering a promising direction to overcome resistance.

Current dual-targeting CAR-T technologies have evolved through multiple innovative approaches, each with distinct technical and clinical advantages. Conventional methods include the co-infusion of two single-target CAR-T products, while co-transduction techniques enable simultaneous expression of two different CAR molecules on individual T cells, allowing synchronized recognition of antigens such as CD19 and CD22. Early clinical trials have preliminarily validated the feasibility and potential of this dual-targeting modality (40). Further refinements in vector design enhance the stability and homogeneity of cellular products: bicistronic vectors encode two CARs within a single transcript, avoiding imbalances associated with multi-vector co-transduction; tandem CAR or loop CAR structures integrate different antigen-binding domains into a single receptor, facilitating synergistic targeting. For instance, a fully humanized bivalent loop CAR-T (CT120), capable of recognizing both CD19 and CD22, has demonstrated promising efficacy in clinical explorations for relapsed/refractory B-cell non-Hodgkin lymphoma (B-NHL) and acute B-lymphoblastic leukemia (B-ALL) (128–130).

The multi-targeting strategy has expanded from dual- to triple- or even higher-order targeting. In B-cell malignancies, trispecific CAR-T cells targeting CD19, CD20, and CD22 simultaneously have been designed to maximize coverage of the tumor antigen spectrum, substantially mitigating relapse risks due to antigen escape (131). Notably, this strategy has further advanced to incorporate “CAR-T plus other immune cell synergy.” For example, CAR-T cells engineered to secrete bispecific NK cell engagers (BiKEs) can target tumor-associated antigens like CD19 or EGFR while recruiting endogenous NK cells via CD16a engagement. This dual design—simultaneously targeting tumors and engaging immune effector cells—has demonstrated superior tumor clearance in heterogeneous tumor models, offering an innovative solution to the challenge of uneven antigen expression (132) (**Table 2**).

**Table 2:** Clinical trials that address antigen-negative escape mechanisms.

ClinicalTrials.gov identifier	Country	Disease		Product	Observations	Refs.
CD19/CD20						
NCT03019055	USA	R/R B cell malignancies		Anti-CD19 and anti-CD20 tandem receptor	PFS 50% and OS 75%	(180)
NCT03097770	China	R/R BCL		Anti-CD19 and anti-CD20 tandem receptor	Best ORR 79%;,CR 71%	(181)
NCT03207178	China	R/R BCL		Co-administration of anti-CD19 and anti-CD20	OS 8.1 month and PFS 5.0 months.	(182)
NCT04792489	USA	R/RDLBCL		CD19/CD20 dual-target CART	NA	NA
NCT04007029	USA	R/R FL, DLBCL, MCL and CLL/SLL		CD19–CD20 bispecific CAR	ORR 90%, CR 70%.	(183)
CD19/CD22						
NCT03620058	USA	R/R B-ALL		CART22-65s with huCART19	CR 74%.	(184)
NCT03233854	USA	R/R B cell malignancies		CD19-22.BB.z	LBCL best ORR 62%; CR 29%.	(185, 186)
AMELIA trial, EUDRA CT 2016-004680-39	UK	Paediatric and young adult patients with relapsed or refractory B-ALL		AUTO3, autologous transduced T cells expressing both anti-CD19 and anti-CD22 CARs	At 1 month after treatment, the remission rate was 86%.The 1-year OS and event-free survival rates were 60% and 32%, respectively	(187)
NCT06798298	USA	R/RDLBCL		a Dual Targeting CAR T Cell Drug Product With a Gene Edit( bbT369)	NA	NA
CD19/CD20/CD22						
NCT05094206	USA	R/R B cell malignancies		CAR20.19.22 T cells	NA	NA
NCT05418088	USA	R/R B cell malignancies		Anti-CD19/CD20/CD22 CAR-T cells	NA	NA
CD19						
NCT02746952	UAS	R/R B cell malignancies	UCART19		ORR 48%,PFS2.1month, os13.4 months。	(188, 189)
Gene therapy						
NCT06528301	USA	R/R large B-cell lymphoma and chronic lymphocytic leukemia	UB-VV111, a gene therapy that generates CD19 CAR T cells in the body.		NA	NA
NCT06539338	USA	R/R BCL	Intravenous injection of a chimeric antigen receptor transgenic specific to CD20 (CAR20) using a lentiviral vector(INT2104)		NA	NA

Beyond basic multi-targeting designs, the development of logic-gated CAR systems introduces an additional layer of precision, enabling “specific recognition + flexible response” advanced strategies. OR-gate CAR-T cells activate upon recognition of any one of two or more target antigens, ideally suited for heterogeneous antigen expression in tumors and effectively blocking escape due to single-antigen loss. Complementarily, AND-gate CAR-T cells require the simultaneous presence of two specific antigens on tumor cells for full activation, significantly enhancing tumor specificity and reducing off-target toxicity against normal tissues. (133–135).

Despite the considerable potential of multi-targeting CAR-T strategies in overcoming antigen escape-mediated resistance, several technical challenges remain. These include potential instability of immune synapses when multiple CAR structures coexist, difficulties in balancing the expression of multiple CAR molecules during vector packaging, and possible host immune responses triggered by immunogenic single-chain variable fragments (scFvs). However, with continued optimization in vector engineering, antigen-binding domain design, and accumulating clinical validation, multi-targeting CAR-T therapies not only provide a powerful technical arsenal against CAR-T resistance but also lay a foundation for future precision immunotherapies targeting complex tumor heterogeneity.

### 3.2 Optimizing CAR-T cells: a multidimensional strategy to overcome therapeutic resistance

Concurrent with multi-targeting approaches to counter antigen escape, multidimensional optimization of CAR-T cells themselves represents a core direction for enhancing their *in vivo* adaptability, cytotoxicity, and persistence to overcome treatment resistance. These optimizations are not isolated interventions but form a systemic efficacy-enhancement framework spanning the entire process from cell sourcing and manufacturing process innovation to genetic engineering and functional modular reinforcement.

The starting material and manufacturing process of CAR-T cells fundamentally determine their functional capacity, serving as the cornerstone of optimization strategies. In patients with hematologic malignancies, autologous T cells are often functionally compromised or terminally differentiated due to disease progression or prior chemotherapy, consequently limiting CAR-T cell activity and proliferative potential. Sourcing T cells from early disease stages or more primitive T-cell subsets (such as bone marrow-infiltrating lymphocytes or naive T cells) yields products with superior expansion capacity (136, 137). The ZUMA-12 trial (NCT03761056) provided clinical validation, demonstrating that CAR-T products manufactured from T cells obtained during first-line therapy contained a higher proportion of naive T cells, which correlated with enhanced *in vivo* expansion (138). Beyond cell sourcing, manufacturing innovations are equally critical. Shortening *ex vivo* culture duration helps preserve native T-cell phenotypes and avoid exhaustion from prolonged expansion, while non-viral-based “rapid manufacturing” platforms not only reduce production costs but also generate more potent non-activated CAR-T cells with significantly enhanced antitumor efficacy (139).

Precise genetic and epigenetic engineering provides key solutions to core challenges including T-cell exhaustion and limited proliferative capacity, thereby enhancing antitumor functionality and durability. To counteract immune checkpoint-mediated inhibition, CRISPR-based knockout of PD-1 or lentiviral co-expression of shRNA targeting both PD-1 and TIGIT can alleviate T-cell exhaustion induced by the tumor microenvironment, with related clinical trials (e.g., NCT04836507) currently exploring this potential (140). Furthermore, genome-wide CRISPR screens have identified additional genetic targets for enhancing CAR-T efficacy; for example, knockout of the cell cycle regulator CDKN1B was shown to augment CAR-T cell proliferation and effector function, significantly improving tumor clearance and overall survival in animal models (141). For allogeneic CAR-T applications, knockout of the glycosylation-regulating gene SPPL3 creates a “glycan shield” that enhances immune tolerance without affecting T-cell receptor function, significantly improving *in vivo* persistence and offering new avenues for off-the-shelf CAR-T therapies (142).

Armored CAR-T engineering, which involves incorporating additional functional modules to overcome inherent biological limitations, represents a crucial pathway for amplifying antitumor potency. To address poor survival and infiltration within the tumor microenvironment, co-expression of cytokines (e.g., IL-12, IL-15) or specific chemokine receptors has emerged as a core strategy. For instance, IL-12-secreting CD19-targeted cord blood-derived CAR-T cells show promise in treating B-cell acute lymphoblastic leukemia (143), while CD19-specific CAR-T cells co-expressing IL-15 and a suicide gene enhance anti-lymphoma/leukemia effects while maintaining a safety switch (144). Additionally, CAR-T cells expressing membrane-bound IL-7 receptors or CCL19 promote homing to and survival within tumor tissues, an approach under investigation in clinical trials such as NCT04381741 (145). To directly counter immunosuppressive mechanisms, armored CAR-T cells can be designed to express a dominant-negative TGF-β receptor to block immunosuppressive signaling, or incorporate metabolic regulatory modules like PIM3 kinase inhibition to reverse hypoxia-induced dysfunction and delay exhaustion (146) (**Table 3**).

**Table 3:** Clinical trials for drug resistance caused by abnormal tumor microenvironment.

ClinicalTrials.gov identifier	Country	Disease	Product	Results	Ref.
NCT04684563	USA	CLL and NHL	huCART19-IL18	NA	NA
NCT04099797	USA	GD2-expressing brain tumours	GD2-specific chimeric antigen and constitutively active IL-7 receptors	NA	NA
NCT03196830	China	R/R lymphoma	Anti-CD30 CAR-T cell treatment combined with an PD1 inhibitor	PFS and OS rates were 45% and 70%, respectively; median follow-up of 21.5 months	(196)
NCT04844086	Taiwan	Advanced lymphoid malignancies	Rapid personalized manufacturing of CD19 mbIL-15 CAR-T cells	Phase I, open-label dose-escalation trial, evaluation of safety and tolerability	NA
NCT03579927	USA	BCL and CLL	CD19-CD28ζ-2A-iCasp9-IL15-transduced cord blood natural killer cells when given together with high-dose chemotherapy and stem cell transplant	Among the 11 patients, 7 achieved complete remission.	NA

In summary, through source control in manufacturing, precision genetic editing, and functional augmentation via armoring, multidimensional optimization achieves a systematic enhancement of CAR-T cell quality and antitumor efficacy. These strategies synergize with multi-targeting approaches, collectively providing a comprehensive solution to overcome CAR-T resistance and advancing the therapy toward greater efficacy and safety.

### 3.3 Remodeling the tumor microenvironment to augment CAR-T cell efficacy

​Following the expansion of CAR-T cell recognition breadth through multi-targeting strategies and the intrinsic optimization of cellular functions, actively reversing the immunosuppressive tumor microenvironment (TME) emerges as a critical step toward achieving durable therapeutic responses. The TME functions as a fortified “stronghold,” where immunosuppressive cells, inhibitory molecules, and signaling pathways collectively impede CAR-T cell infiltration and cytotoxicity. Consequently, deliberate modulation of this milieu represents a pivotal avenue for deepening antitumor efficacy.

Neutralizing immunosuppressive components within the TME constitutes the primary intervention for rescuing CAR-T cell function. Immunosuppressive cells (e.g., regulatory T cells, myeloid-derived suppressor cells) and inhibitory cytokines (e.g., TGF-β, IL-10) form the core elements of this barrier. Pharmacologic depletion or functional inhibition of these subsets directly mitigates their suppressive pressure on CAR-T cells, while neutralizing antibodies against inhibitory cytokines remove functional “roadblocks” to T-cell activation and infiltration. Preclinical studies confirm that blocking IL-10 signaling significantly enhances antitumor immunity in models of colorectal cancer, providing robust validation for this approach (122).

Combination with immune checkpoint inhibitors represents another widely explored strategy. Aberrant activation of checkpoint pathways (e.g., PD-1, LAG-3, TIM-3) is a key mechanism of CAR-T cell suppression in the TME, addressed through both exogenous drug combinations and endogenous genetic engineering. Co-administering checkpoint inhibitors post CAR-T infusion effectively “releases” inhibited CAR-T and endogenous T cells, restoring their antitumor capacity (147). Ongoing clinical trials such as the PLATEAU study (NCT06290622) are evaluating triple blockade of PD-1, LAG-3, and TIM-3 in patients with DLBCL who failed CAR-T therapy. Beyond exogenous combinations, genetic engineering empowers CAR-T cells with autonomous resistance to suppression; examples include dominant-negative PD-1 receptors to block inhibitory signaling, secretion of anti-PD-1 scFv fragments for localized checkpoint blockade (148, 149), and sophisticated PD-1–CD28 chimeric switch receptors that reprogram inhibitory signals into activating signals (150), thereby enhancing CAR-T cell activation upon encountering PD-L1 (151, 152) (**Table 4**).

**Table 4:** Clinical trials for abnormal CART function.

ClinicalTrials.gov identifier	Country	Disease	Construct	Summary of results (if available)
PD1–PDL1 blockade				
NCT03044743	China	EBV-associated malignancies (DLBCL, among others)	EBV cytotoxic lymphocytes	NA
NCT04163302	China	BCL	Anti-CD19 CAR-T cell	NA
NCT03298828	China	B cell malignancies	Anti-CD19 CAR-T cell	NA
NCT04539444 (192)	Taiwan	BCL	Anti-CD19/CD22 CAR-T cell	ORR 87.5% and CR 68.8%.
NCT03258047 (193)	China	BCL	Anti-CD19 CAR-T cell	CR 56.4%, PR 23.1%.
NCT04381741	China	DLBCL	Anti-CD19 CAR-T cell	NA
PD1–TIGIT blockade				
NCT04836507 (140)	Republic of Korea	LBCL	Anti-CD19 CAR-T cell	NA
Combining CAR therapy with signal transduction inhibition via BTK-dependent and BTK-independent mechanisms				
NCT02640209 (194)	USA	CLL/SLL	Anti-CD19 CAR-T cell	Adding autologous anti-CD19 humanized binding domain T cells to ibrutinib in patients with CLL not in complete remission led to deep and durable remissions
NCT03331198 (195)	USA	CLL/SLL	Anti-CD19 CAR-T cell	Administration of ibrutinib in combination with CAR-T cells was well tolerated and might decrease the incidence of severe cytokine release syndrome and improve responses

Small-molecule drugs, with their diverse mechanisms and favorable penetration profiles, serve as versatile tools for TME modulation. Bruton’s tyrosine kinase (BTK) inhibitors (153) and demethylating agents synergize (154) with CAR-T cells in clinical studies, with the latter also enhancing persistence through epigenetic reprogramming (155). Apoptosis-targeting agents such as BCL-2 inhibitors (55) and IAP antagonists (156) lower the threshold for tumor cell death, augmenting CAR-T–mediated killing. Epigenetic modulators including HDAC inhibitors (157) and EZH2 inhibitors (158) improve CAR-T efficacy by upregulating tumor antigen expression and reshaping immune contexture. Additionally, PI3K-AKT inhibitors promote central memory T-cell differentiation, enhancing longevity (159, 160), while Toll-like receptor agonists recruit immune cells to tumor sites (161), and immunomodulatory drugs like lenalidomide fine-tune T-cell function to improve overall immune responses.

Notably, recent interventions targeting metabolic dysregulation in the TME show promising potential. Metabolic waste products such as adenosine and kynurenine directly suppress T-cell function via specific receptors. A2AR antagonists blocking adenosine signaling (162) and IDO inhibitors reducing kynurenine production have both been demonstrated to improve CAR-T cell function and persistence (163, 164). Furthermore, abnormal tumor vasculature causing hypoxia and disordered permeability creates a physical barrier to CAR-T cell infiltration; vascular normalizing agents like anti-VEGFR2 antibodies alleviate hypoxia and promote CAR-T cell trafficking, offering novel strategies to overcome physical barriers (165).

Through these multi-layered, mechanistically diverse interventions, the immunosuppressive TME can be remodeled into a supportive niche conducive to CAR-T cell activity. As our understanding of the TME deepens, more precise and potent combination strategies will undoubtedly emerge, ultimately improving treatment outcomes for patients (**Table 5**).

**Table 5:** Clinical trials targeting the mechanism of antigen-positive drug resistance.

ClinicalTrials.gov identifier	Country	Disease	Construct
NCT03331198 (190)	USA	R/R CLL or SLL	JCAR017 (lisocabtagene maraleucel) + venetoclax
NCT03310619 (1)	USA	Aggressive B cell NHL	JCAR017 (lisocabtagene maraleucel) + ibrutinib
NCT03876028	USA	R/R DLBCL lymphoma	Tisagenlecleucel in combination with ibrutinib
NCT02650999 (191)	Republic of Korea	R/R DLBCL lymphoma	Tisagenlecleucel in combination with Pembrolizumab
NCT04257578	USA	BCL	Axicabtagene ciloleucel in combination with acalabrutinib

### 3.4 Combination therapies and novel platforms: synergistic strategies to overcome CAR-T resistance

In the ongoing pursuit to overcome resistance in CAR-T therapy, combination strategies leveraging synergistic mechanisms and novel regulatable platforms capable of precise functional control have emerged as two pivotal directions to break through existing efficacy bottlenecks. These innovative approaches not only enhance antitumor activity through multi-mechanism cooperation but also optimize safety profiles via intelligent regulatory technologies, offering promising alternatives for patients with refractory disease.

The combined application of bispecific antibodies (BsAbs) — particularly bispecific T-cell engagers (BiTEs) — with CAR-T cells represents a significant advancement by activating endogenous immune-killing pathways and compensating for CAR-T recognition limitations. This strategy addresses the shortcomings of monotherapies while amplifying joint tumoricidal efficacy. BsAbs simultaneously bind CD3 on T cells and tumor-associated antigens, thereby bridging endogenous T cells with malignant cells to activate an additional killing route. Such a combination not only compensates for CAR-T blind spots caused by antigen downregulation but also broadens the spectrum of tumor elimination through synergistic effects. For instance, combining CD3×CD20 BsAbs with CD19-CAR-T in relapsed/refractory B-cell non-Hodgkin lymphoma has demonstrated a dual mode of action: BsAbs mediate initial debulking by bridging immune cells, while low-dose BsAb maintenance after CAR-T infusion promotes synergistic antitumor immunity and enhances *in vivo* expansion and persistence of CAR-T cells. Moreover, BsAbs have been shown to promote T-cell differentiation toward a central memory phenotype and reduce exhaustion markers (e.g., PD-1, TIM-3, LAG-3), thereby reinforcing CAR-T functional durability (166). Similarly, combining CAR-T with BiTEs offers unique advantages; BiTEs can activate regulatory T cells to release perforin and granzymes, forming a dual-targeting killing mode with CAR-T that improves clearance of resistant tumor cells (167). This “CAR-T targeted killing plus BiTE-activated endogenous immunity” model effectively addresses resistance scenarios caused by tumor heterogeneity, offering a new paradigm for combination therapy.

The clinical utility of CAR-T cells has been limited by tonic signaling-induced exhaustion and off-target toxicities. Novel regulatable CAR systems incorporating externally controllable “switch” mechanisms enable precise control over CAR-T activation, mitigating persistent activation-driven resistance while flexibly addressing safety risks. Among these, pharmacologically gated switches based on protease/inhibitor regulation are relatively well-established, operating primarily through ON-switch and OFF-switch modes (168). ON-switch CARs maintain structural integrity and propagate activation signals only in the presence of specific small-molecule inhibitors, achieving “drug-dependent activation”; conversely, OFF-switch CARs dissociate their bipartite structure upon inhibitor addition, rapidly terminating cytotoxic activity. The core advantage of such switchable platforms lies in their on-demand controllability: initial switch activation concentrates antitumor efficacy during peak tumor burden, while deactivation induces a quiescent state when toxicity emerges or tumor load declines. This dynamic regulation averts exhaustion caused by persistent tonic signaling and significantly widens the therapeutic window, balancing efficacy and safety (169).

In summary, combination therapies and novel platforms are advancing CAR-T technology multidimensionally. Integrating BsAbs/BiTEs and pharmacologically precise switches not only expands the breadth and depth of CAR-T–mediated killing but also effectively manages toxicity and exhaustion dynamics. With the emergence of more intelligent circuit designs and target resources, these innovations are poised to systematically overcome resistance, offering durable and effective treatment options for a broader patient population (**Table 6**).

**Table 6:** Strategies to overcome resistance to CAR-T cell therapy in DLBCL.

Strategy	Specific approach	Research focus	Outcome/mechanism	Refs
1. Preventing Antigen Escape	Multi-Targeting CARs	Dual-targeting CARs(e.g.,CD19/CD22)	Reduces relapse risk from single antigen loss	(134)-(137)
		Tri-specific CARs (e.g., CD19/CD20/CD22)	Maximizes antigen coverage to minimize escape	(138)
	Immune Cell Recruitment	CAR-T cells secreting BiKEs (recruits NK cells)	“Targeting + recruitment” dual-killing of heterogeneous tumors	(139)
	Logic-Gated CARs	“OR”-gate CARs	Activation upon recognition of any one antigen; prevents escape from single antigen loss	(140) (141) (142)
		“AND”-gate CARs	Full activation only upon co-recognition of two antigens; enhances tumor specificity and reduces on-target/off-tumor toxicity	(140) (141)] (142)
2. Optimizing CAR-T Cells	Source & Manufacturing	Optimal T-cell source (e.g., naive T cells)	Generates CAR-T products with superior proliferative potential and reduced exhaustion	(67) (143) (144),,
		Improved manufacturing (e.g., shortened culture)	Preserves a less differentiated T-cell phenotype, enhancing antitumor effect	(145)
	Gene Editing	Knockout of immune checkpoints (e.g., PD-1, TIGIT)	Alleviates T-cell exhaustion induced by the TME	(146)
		Knockout of functional genes (e.g., CDKN1B, SPPL3)	Enhances CAR-T proliferation, effector function, and persistence	(147) (148),
	Armored CAR-T	Co-expression of cytokines (e.g., IL-12, IL-15)	Improves CAR-T survival and function within the TME	(149) (150),
		Co-expression of chemokine receptors (e.g., CCL19)	Promotes T-cell homing to and retention within tumor sites	(151)
		Expression of dominant-negative TGF-β receptor	Blocks TGF-β-mediated immunosuppressive signaling	(152)
3. Controlling Tumor Microenvironment (TME)	Targeting Suppressive Components	Depleting immunosuppressive cells (e.g., Tregs, MDSCs)	Directly削弱 (weakens) suppression on CAR-T cells	(153)
		Neutralizing inhibitory cytokines (e.g., TGF-β, IL-10)	Removes barriers to CAR-T cell infiltration and function	(153)
	Overcoming Checkpoint Inhibition	Combination with checkpoint inhibitors (e.g., anti-PD-1/LAG-3)	Reinvigorates suppressed CAR-T and endogenous T cells	(154)
		Genetic modification (e.g., PD-1–CD28 switch receptor)	Reprograms inhibitory signals into activating signals	(155)–(159)
	Combination with Small Molecules	BTK inhibitors	Synergistic tumor clearance	(160)
		Demethylating agents	Synergistic tumor clearance and enhanced CAR-T persistence via epigenetic reprogramming	(161) (162),
		BCL-2 inhibitors, IAP antagonists	Lowers tumor cell survival threshold, enhancing CAR-T killing efficiency	(59) (163),
		HDAC inhibitors, EZH2 inhibitors	Enhances tumor antigen expression and remodels the immune microenvironment	(164) (165),
		PI3K-AKT inhibitors	Promotes central memory T-cell differentiation, enhancing persistence	(166) (167),
		TLR agonists, Lenalidomide	Promotes immune cell recruitment and modulates T-cell function via multiple pathways	(168)
	Metabolic & Vascular Intervention	A2AR antagonists, IDO inhibitors	Improves CAR-T function and persistence in the metabolic TME	(169)– (171)
		Anti-VEGFR2 antibody (vessel normalization)	Alleviates hypoxia and promotes CAR-T cell infiltration	(172)
4. Novel Platforms & Combinations	Combination with BsAb/BiTE	Combination with Bispecific Antibodies (e.g., CD3-CD20)	Compensates for CAR-T recognition gaps; engages endogenous T cells	(173)
		Combination with BiTE (Bispecific T cell Engagers)	Enables dual-targeting killing, improving clearance of resistant cells	(174)
	Controllable CAR Systems	ON/OFF-switch drug-gated CARs	Allows precise, on-demand control of CAR-T activity; enhances safety and prevents exhaustion	(175) (176),

This table categorizes and summarizes the primary research strategies being investigated to overcome mechanisms of resistance to CAR-T cell therapy. The strategies are divided into four main pillars: preventing antigen escape, intrinsic optimization of CAR-T cells, modulation of the tumor microenvironment (TME), and the development of novel platforms and combination therapies.

CAR, Chimeric Antigen Receptor; TME, Tumor Microenvironment; BiKE, Bispecific NK cell Engager; BiTE, Bispecific T cell Engager; BsAb, Bispecific Antibody; TGF-β, Transforming Growth Factor Beta; Treg, Regulatory T Cell; MDSC, Myeloid-Derived Suppressor Cell; A2AR, Adenosine A2A Receptor; IDO, Indoleamine 2,3-Dioxygenase; VEGFR2, Vascular Endothelial Growth Factor Receptor 2; HDAC, Histone Deacetylase; IAP, Inhibitor of Apoptosis Protein; TLR, Toll-Like Receptor; TCR, T-cell Receptor; scFv, single-chain variable fragment.

## 4 Conclusion

Resistance of diffuse large B-cell lymphoma (DLBCL) to chimeric antigen receptor T-cell (CAR-T) therapy constitutes a systematic defense network involving complex interactions among tumor cells, effector T cells, and the tumor microenvironment (TME). Recent research has progressively revealed that these mechanisms exhibit highly heterogeneous regulatory patterns across different molecular subtypes of DLBCL. This complexity is particularly exemplified by T-cell/histiocyte-rich large B-cell lymphoma (THRLBCL). This subtype is histologically characterized by scarce malignant B-cells dispersed within an extensive infiltrate of reactive lymphocytes, concurrently exhibiting widespread constitutive PD-L1 overexpression (45, 170), These features collectively contribute to impaired CAR-T cell recognition and rapid functional exhaustion. Clinical studies indicate that THRLBCL patients receiving CAR-T monotherapy face a two-year cumulative relapse/progression rate as high as 69% (171), whereas combination with PD-1 inhibitors can achieve long-term sustained remission (172). This compellingly demonstrates the critical importance of combined immune checkpoint blockade in this patient population (173).

Beyond THRLBCL, other DLBCL subtypes also demonstrate distinct resistance profiles: primary mediastinal large B-cell lymphoma (PMBL) utilizes 9p24.1 amplification to concurrently drive PD-L1 overexpression and JAK-STAT pathway activation (174, 175); double-hit/triple-hit lymphoma (DHL/THL) relies on BCL-2 overexpression and MYC-driven TME remodeling (176, 177); and EBV-positive DLBCL induces PD-L1 overexpression through LMP1-mediated NF-κB pathway activation (178). These subtype-specific resistance patterns suggest that combining targeted agents—such as immune checkpoint inhibitors, JAK inhibitors, or BCL-2 inhibitors—with CAR-T therapy may represent an effective strategy to overcome resistance across these distinct entities.

Future research should focus on several key dimensions to overcome resistance in CAR-T therapy. A deeper dissection of the molecular mechanisms underlying resistance is essential, particularly the dynamic interplay between tumor cells and the immune microenvironment, as well as the regulatory role of epigenetic modifications in CAR-T cell exhaustion. Subsequently, developing predictive models for resistance leveraging single-cell multi-omics and spatial transcriptomics will be crucial. Such models can finely delineate the cellular and molecular atlas of resistant microenvironments across different DLBCL subtypes, ultimately facilitating personalized treatment strategies and improving the accuracy of efficacy prediction (179). Furthermore, advancing novel CAR-T technologies—including allogeneic (off-the-shelf) CAR-T, regulatable CAR-T, and dual-epitope targeting CAR—holds promise for addressing persistent challenges like antigen escape and immunogenicity. Lastly, conducting large-scale, multi-center prospective clinical trials is imperative to systematically evaluate the efficacy and safety of innovative combination strategies, thereby accelerating the translation of basic research findings into clinical applications.

With the deepening understanding of resistance mechanisms to CAR-T therapy in DLBCL and the continuous innovation of therapeutic strategies, we are poised to overcome current therapeutic bottlenecks, offering patients more effective and durable treatment options, ultimately improving long-term survival and quality of life for DLBCL patients.

## Glossary

CAR-T: Chimeric antigen receptor T-cell
DLBCL: Diffuse large B-cell lymphoma
TME: Tumor microenvironment
scFv: Single-chain variable fragment
ITAM: Immunoreceptor tyrosine-based activation motif
LOH: Loss of heterozygosity
ER: Endoplasmic reticulum
ERAD: Endoplasmic reticulum-associated degradation
APOBEC3: Apolipoprotein B mRNA editing enzyme; catalytic polypeptide-like 3
DNMT3A: DNA methyltransferase 3A
H3K27me3: Histone H3 lysine 27 trimethylation
EZH2: Enhancer of zeste homolog 2
VEGF-A: Vascular endothelial growth factor A
STAT6: Signal transducer and activator of transcription 6
UPR: Unfolded protein response
IFITM1: Interferon induced transmembrane protein 1
B-ALL: B-cell acute lymphoblastic leukemia
IFN-γ: Interferon-gamma
PD-1: Programmed cell death protein 1
PD-L1: Programmed death-ligand 1
ITIM: Immunoreceptor tyrosine-based inhibitory motif
SHP2: Src homology region 2-containing protein tyrosine phosphatase 2
TGF-β: Transforming growth factor β
CTLA-4: Cytotoxic T-lymphocyte-associated protein 4
LAG3: Lymphocyte-activation gene 3
TIM-3: T-cell immunoglobulin and mucin-domain containing-3
PLCγ1: Phospholipase C gamma 1
IRF4: Interferon regulatory factor 4
BATF: Basic leucine zipper ATF-like transcription facto
CD47: Cluster of differentiation 4
SIRPα: Signal-regulatory protein alpha
TNF-α: Tumor necrosis factor alpha
TNFR1: Tumor necrosis factor receptor 1
STAT1: Signal transducer and activator of transcription 1
Kd: Dissociation constant
MHC: Major histocompatibility complex
FcγR: Fc gamma receptor
Teff: Effector T cell
Tscm: Stem-like memory T cell
TMD: Transmembrane domain
ROS: Reactive oxygen species
AICD: Activation-induced cell death
PI3K: Phosphoinositide 3-kinas
AKT: Protein kinase B (PKB)
mTOR: Mechanistic target of rapamycin
TRAF2: TNF receptor associated factor
NF-κB: Nuclear factor kappa-light-chain-enhancer of activated B cells
CPT1A: Carnitine palmitoyltransferase 1A
MAPK/ERK: Mitogen-activated protein kinase/Extracellular signal-regulated kinas
FOXO1: Forkhead box protein O1
Ac-CoA: Acetyl-Coenzyme A
GZMB: Granzyme
TET2: Tet methylcytosine dioxygenase 2
DNMT: DNA methyltransferase
AP-1: Activator protein 1
JUN: Jun proto-oncogene
IRF: Interferon regulatory factor
bZIP: Basic leucine zipper
TOX: Thymocyte selection-associated high mobility group box protein
NR4A: Nuclear receptor subfamily 4 group
BET: Bromodomain and extra-terminal motif
BRD2/4: Bromodomain containing protein 2/4
ID3: Inhibitor of DNA binding 3
SOX4: SRY-box transcription factor 4
IRF8: Interferon regulatory factor 8
PAX5: Paired box 5
TNFRSF14: Tumor necrosis factor receptor superfamily member 14
HVEM: Herpesvirus entry mediator
TMEM30A: Transmembrane protein 30A
Tregs: Regulatory T cells
MDSCs: Myeloid-derived suppressor cells
TAMs: Tumor-associated macrophages
IL-2: Interleukin-2
IL-2Rα: Interleukin-2 receptor alpha chain
ARG1: Arginase 1
CCL22: C-C motif chemokine ligand 22
GLUT1: Glucose transporter 1
IDO: Indoleamine 23-dioxygenase
GCN2: General control nonderepressible 2 kinas
eIF2α: Eukaryotic initiation factor 2 alpha
mTORC1: Mechanistic target of rapamycin complex 1
CD39: Cluster of differentiation 39
CD73: Cluster of differentiation 73
ATP: Adenosine triphosphate
cAMP: Cyclic adenosine monophosphate
PKA: Protein kinase A
ECM: Extracellular matrix
FAK: Focal adhesion kinase
HA: Hyaluronic acid
CASCs: Cancer-associated stromal cells
HSPGs: Heparan sulfate proteoglycans
VCAM-1: Vascular cell adhesion molecule 1
ICAM-1: Intercellular adhesion molecule
CXCL9/10: C-X-C motif chemokine ligand 9/10
CXCR4: C-X-C chemokine receptor type 4
CAFs: Cancer-associated fibroblasts
FAP: Fibroblast activation protein
Cx43: Connexin 43
IL-10: Interleukin-10
PGE2: Prostaglandin E2
CRS: Cytokine release syndrome
ICANS: Immune effector cell-associated neurotoxicity syndrome
GCs: Glucocorticoids
CCR7: C-C chemokine receptor type
STAT3: Signal transducer and activator of transcription
UPS: Ubiquitin-proteasome system
BTK: Bruton’s tyrosine kinase
HDAC: Histone deacetylase
IAP: Inhibitor of apoptosis protein
OS: Overall survival.
